# Benchmarking dynamic analysis methods in diffusion-based single-molecule FRET

**DOI:** 10.1016/j.crmeth.2026.101529

**Published:** 2026-07-14

**Authors:** Joel A. Crossley

**Affiliations:** 1 Astbury Centre for Structural Molecular Biology, School of Molecular and Cellular Biology, Faculty of Biological Sciences, University of Leeds, Leeds LS2 9JT, UK

**Keywords:** single-molecule, FRET, smFRET, conformational dynamics, benchmarking, kinetics, fluorescence, hidden Markov model, biophysics, structural biology

## Abstract

Single-molecule Förster resonance energy transfer (smFRET) provides nanometer-scale snapshots of biomolecular conformational dynamics, but extracting reliable dynamic information from diffusion-based measurements requires a careful choice of analysis method. This study benchmarks the widely used approaches, including qualitative indicators of dynamics and quantitative rate extraction via hidden Markov modeling, using simulated datasets spanning common experimental conditions and focusing on a two-state system. The assessment clarifies the timescales each method reports on, determines the number of bursts required for reliable inference, examines the differences in FRET efficiencies needed by each approach, and evaluates the accuracy of kinetic rate recovery. Together, these results provide practical guidelines for selecting analysis strategies and accurately interpreting conformational dynamics in diffusion-based smFRET. The accompanying datasets offer a resource for optimizing experimental design and developing new analytical methods.

## Highlights

• Benchmarks dynamic analysis methods for diffusion-based single-molecule FRET
• Defines the kinetic regimes accessible to each analysis approach
• Quantifies how state separation, populations, and dataset size affect sensitivity
• Assesses kinetic rate recovery using photon-by-photon hidden Markov modeling

## Motivation

Diffusion-based single-molecule Förster resonance energy transfer (smFRET) experiments detect bursts of fluorescence as individual labeled biomolecules transiently enter the observation volume, providing snapshots of their conformational dynamics. Several analysis methods exist for interpreting these measurements, each with different sensitivities to kinetic timescales, state separation, and dataset size, yet their relative performance has not been systematically characterized. As smFRET adoption grows beyond specialist laboratories, such a characterization is increasingly valuable. This study systematically benchmarks the commonly used dynamic analysis approaches using simulated datasets representative of typical experimental conditions, providing practical guidelines for analyzing conformational dynamics in diffusion-based smFRET.

## Graphical abstract

**Figure:**
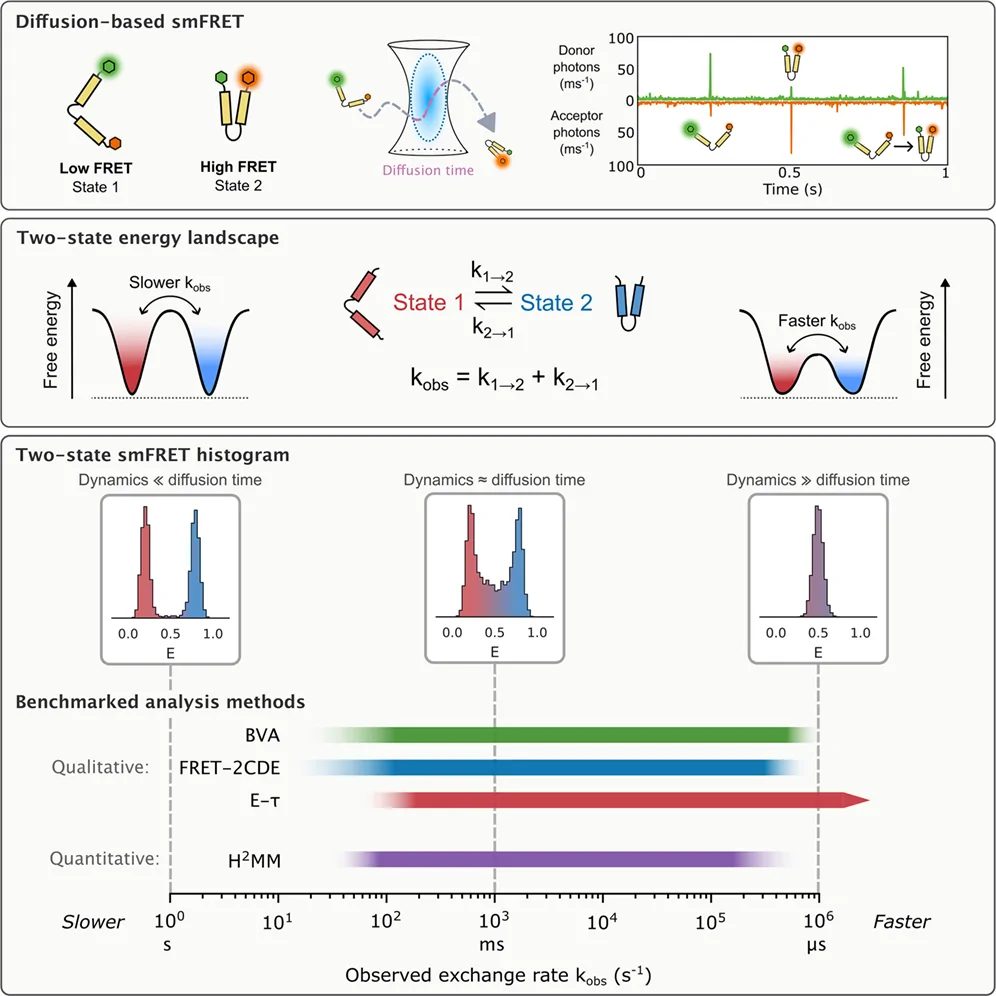

## Teaser

Crossley benchmarks widely used dynamic analysis methods for diffusion-based smFRET using simulated datasets, clarifying the timescales, burst number requirements, and FRET state separation each approach demands. The results offer practical guidelines for selecting analysis strategies and accurately interpreting conformational dynamics in biomolecules.

## Introduction

Over the past two decades, single-molecule Förster resonance energy transfer (smFRET) has rapidly evolved from a niche field of experimental biophysics, accessible only to experts in specialized optical laboratories, into a readily accessible technique for many researchers across many disciplines.^1^^,^^2^^,^^3^^,^^4^^,^^5^ Many commercial microscopes commonly found in imaging facilities offer all the hardware needed to acquire data, and a plethora of community-built open-source software for analysis is available. As a result, the primary barrier to entry has shifted from the technical challenge of data collection to the conceptual challenge of choosing among a diverse set of experimental approaches and analysis methods and their correct interpretation. This manuscript aims to guide newcomers to diffusion-based smFRET by outlining the strengths and limitations of commonly used analysis approaches. Comparative insights into the timescales each method can access are provided along with a discussion on how these align with the dynamics of biologically relevant systems. It is important to clarify that the goal of this manuscript is not to declare a single analysis method as superior. Rather, it seeks to highlight the contexts in which different approaches are most appropriate and what types of information they can yield.

**Figure 1:**
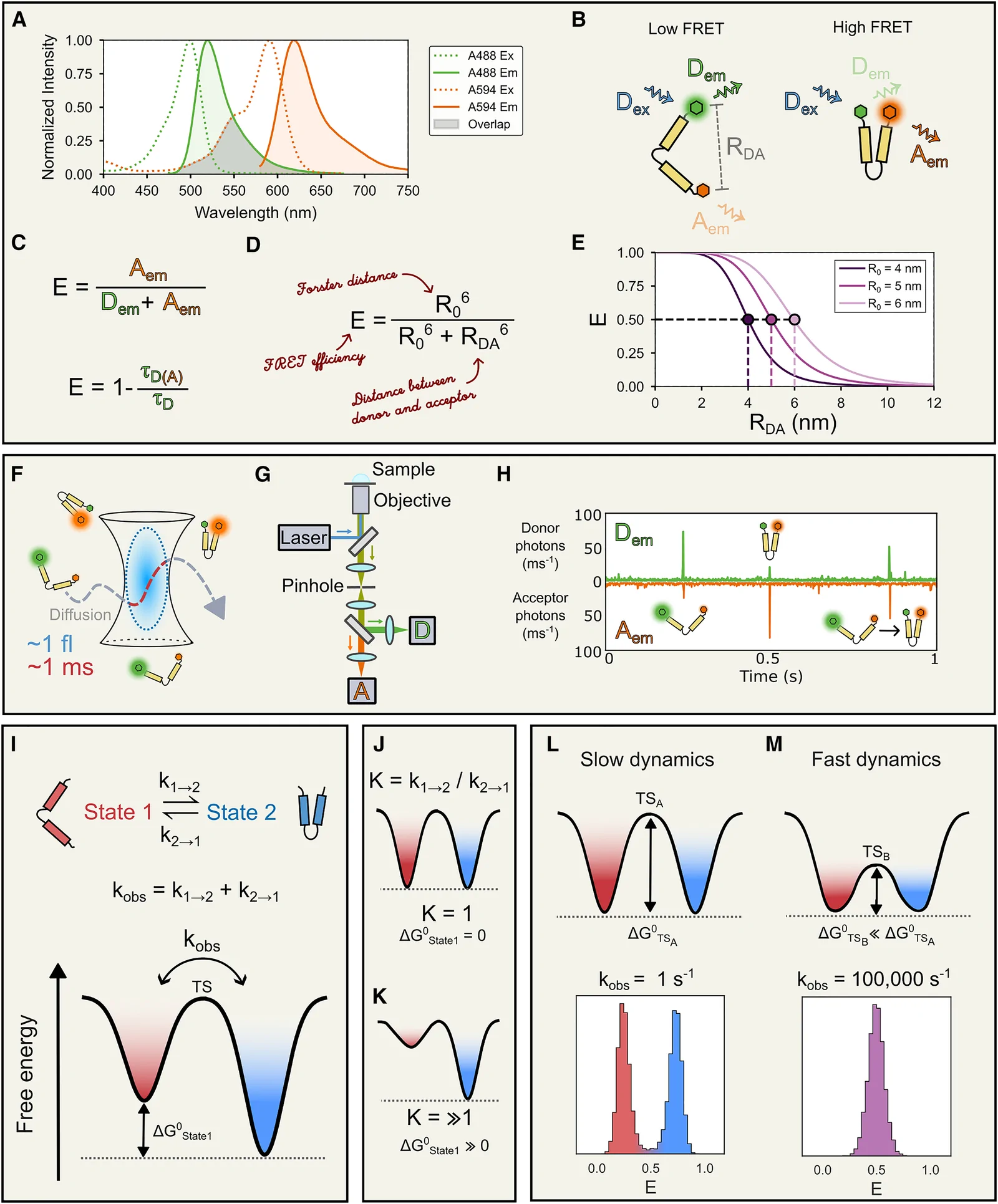
Conceptual framework for understanding FRET, diffusion-based smFRET, and relative populations and dynamics between two conformational states using smFRET (A) Excitation (ex) and emission (em) spectra of a donor and acceptor fluorophore. (B) A donor-and-acceptor-labeled biomolecule (light yellow) in a low-FRET state (left) when the distance between the fluorophores (R_DA_) is high and energy transfer between them is low and a high-FRET state (right) when they are close together and the efficiency of energy transfer is high. (C) Equations for calculation of E by fluorescence intensities (top) or the excited-state lifetime of the donor (bottom). (D) Equation for calculation of E from R_DA_ and the Förster distance (R_0_). (E) Changes in Förster distance (R_0_; 4, 5, and 6 nm) translate into the distance-dependent efficiency curves. The distances that yield a FRET value of 0.50 are shown in dashed lines for each curve. (F) Illustrative confocal volume with a single molecule diffusing in and out of the ∼1-fL detection volume with a diffusion time of ∼1 ms. (G) Schematic of a basic confocal microscope used for diffusion-based smFRET. (H) Example smFRET time trace showing the donor and acceptor fluorophore emission in green and orange, respectively. (I) A two-state conformational exchange between an extended state (State 1; red) and a compact state (State 2; blue). (J) The equilibrium constant K determines the relative depth of the wells. (K) When K ≫ 1 then ΔG°_State1_ ≫ 0 and State 1 is sparsely populated. (L) Barrier height controls the rate of dynamic interchange between states. When the transition-state barrier is high (TS_A_), dynamics are slow relative to the diffusion time through the confocal volume, and clear separation of the high-FRET (blue) and low-FRET (red) states is observed in the histogram (bottom). (M) A low transition-state barrier (TS_B_) produces dynamics that are fast relative to the diffusion time, leading to a single averaged FRET distribution (purple).

FRET occurs when the emission spectrum of an excited donor fluorophore overlaps with the absorption/extinction spectrum of the acceptor (Figure 1A), allowing for non-radiative dipole-dipole coupling. The efficiency of FRET (E) is inversely proportional to the distance between the fluorophores (R_DA_); therefore, fluorophores can be conjugated to the biomolecules of interest and E can be used as a “spectroscopic ruler” to probe the conformation and dynamics (Figure 1B). FRET efficiency can be calculated directly from the fluorescence intensities of the donor and acceptor or the excited state lifetime of the donor in the absence (*τ*_D_) and presence of the acceptor (*τ*_D(A)_) (Figure 1C). As E scales inversely with the sixth power of R_DA_^6^ (Figure 1D), FRET can be used as a spectroscopic ruler over donor-acceptor distances of ∼2–10 nm (Figure 1E).

smFRET can be implemented using various approaches, with the two most common being (1) surface-immobilized (using widefield, or more commonly total internal reflection fluorescence [TIRF], microscopy with camera-based detection) and (2) diffusion-based (Figure 1F) (confocal microscope setup with single-photon counting detectors [Figure 1G]). This work focuses on the latter. Diffusion-based techniques have gained popularity in recent years in part due to their ease of use, eliminating the need for complex biomolecule tethering protocols, as well as their ability to probe more rapid timescales than TIRF. Moreover, diffusion-based approaches inherently avoid potential artifacts arising from surface interactions, as the biomolecules remain freely diffusing in solution rather than being tethered to a substrate. In this regime, individual molecules are detected as bursts of fluorescence that typically last ∼1 ms (Figure 1H). This manuscript will refer to diffusion-based smFRET as just smFRET hereafter, unless stated otherwise. A recent comparative blind study among 20 laboratories using smFRET demonstrated a high degree of precision and accuracy in measurements of non-dynamic FRET samples.^7^ A follow-up study further confirmed the technique’s reliability for investigating conformational dynamics in proteins.^8^ For a recent benchmark study comparing analysis tools for surface-immobilized camera-based measurements, the reader is referred to Götz et al.^9^ (the typical time resolution of these types of experiment is upward of ∼10–100 ms).

The choice between these two approaches depends strongly on the properties of the system under study and the timescales of interest. Surface-immobilized measurements enable prolonged observation of individual molecules over seconds to minutes, making them ideal for studying slow (>∼10 ms) conformational changes,^10^ multi-step enzymatic cycles,^11^ or binding and unbinding events that unfold over long timescales.^12^ However, immobilization requires specific tethering chemistries that must be carefully validated to avoid surface-induced artifacts and can be particularly challenging for intrinsically disordered proteins or systems where surface proximity may perturb the conformational ensemble. Diffusion-based approaches, typically implemented using confocal detection, instead observe freely diffusing molecules as they traverse the detection volume, providing sensitivity to faster dynamics on sub-millisecond to millisecond timescales. This makes them well suited for studying rapid conformational exchange and systems where surface attachment is impractical or undesirable. The trade-off is that individual observation times are limited to the burst duration, precluding direct observation of slow processes from single trajectories. In practice, the two approaches, therefore, occupy complementary regimes: surface-immobilized measurements excel at tracking slow, sequential processes with long observation windows, whereas diffusion-based measurements are better suited for characterizing rapid equilibrium fluctuations free from surface effects. The appropriate choice is, therefore, system dependent and guided by the specific biological question being addressed, and in some cases, the two approaches can provide complementary insight into the same conformational landscape.

Protein function is often governed by transitions between discrete conformational states. In the case of two states, this can be conceptualized as movement across a simple energy landscape^13^^,^^14^ (Figure 1I), with each state represented with a well where a barrier between them (i.e., the transition state [TS]) dictates the rates of interconversion between the states (i.e., k_1→2_ and k_2→1_ or k_12_ and k_21_) with a total rate of interconversion k_obs_. While rates of interconversion vary, the barrier crossing itself, often referred to as the transition path time, takes less than 1 μs.^15^ The depth of the well reflects the standard Gibbs free energy (G°) of that state; the deeper the well, the more stable the state. The relative difference in the depth of the wells (ΔG°), and the rate of interconversion between them, therefore, dictates the equilibrium constant (K). When k_12_ = k_21_ then ΔG°_State1_ = 0 and K = 1 (Figure 1J); when k_12_ > k_21_ then ΔG°_State1_ > 0 and K > 1 (Figure 1K). When the ΔG° of the TS is high (Figure 1L; TS_A_), then k_obs_ is slow relative to the residence time of single molecules in the confocal volume and a clear separation of FRET populations can be observed. When the ΔG° of TS is low (Figure 1M; TS_B_) and the rate of k_obs_ is much greater than the diffusion time, then dynamic averaging of the FRET populations occurs due to the high probability that a transition will occur during the observation.

In this fast regime, FRET efficiency histograms cannot be translated into the underlying energy landscape (Figure 1M), so reliable conclusions hinge on analysis techniques that account for the dynamics between FRET states, and it is imperative to define the contributions of such dynamics to the E distributions to draw correct conclusions from the data. To guide such research, quantitative benchmarking of single-molecule FRET analysis methods is required so that the correct suite of smFRET experiments can be conducted and the data thereby correctly analyzed. Providing a benchmark so that this can be readily performed by experienced users and novices to smFRET alike is the objective of this manuscript.

A current list of commonly used analysis techniques to extract dynamics from individual single-molecule events can be found in Table 1, and a visualization of how they work can be found in Figure S1. In brief, burst variance analysis (BVA) subdivides each burst into small windows of photons and calculates the standard deviation of FRET efficiency across these windows (σ_E_); a σ_E_ exceeding that expected from shot noise alone indicates that the FRET efficiency changed during the burst (Figure S1A). Burst-wise fluorescence lifetime (E-τ) compares the FRET efficiency of each burst with the normalized mean donor fluorescence lifetime (τ_D_), where a static molecule is expected to follow a well-defined linear relationship between the two. Deviations from this relationship indicate that the observed FRET efficiency reflects an average over multiple states visited during the burst (Figure S1B). FRET two-channel kernel-based density distribution estimation (FRET-2CDE) uses kernel density estimation to assess the local ratio of acceptor to donor photon density around each photon throughout the burst. For a static molecule, these local estimates converge to the burst average, yielding a low FRET-2CDE score; when transitions occur during the burst, systematic shifts in local photon densities produce a higher score, indicating dynamics (Figure S1C). For each of these methods, the expected relationship for bursts exhibiting no dynamics on the observation timescale can be calculated and is often plotted as a “static line,” against which experimental data are compared. Photon-by-photon hidden Markov modeling (H^2^MM) takes a different approach, operating directly on the sequence of photon arrival times and their channel assignments to fit a hidden Markov model via maximum likelihood estimation. This enables the extraction of rate constants for transitions between FRET states, along with the FRET efficiencies of the underlying states themselves (Figure S1D). In the following sections, these approaches are benchmarked using datasets simulating dynamics between two FRET states to establish the conditions under which each can reliably detect and quantify conformational dynamics.

**Table 1:** Overview of commonly used current methods to detect and quantify conformational dynamics in diffusion-based single-molecule FRET experiments

Analysis method	Abbreviation	Brief description	Reference
Burst variance analysis	BVA	Splits bursts in selections of photons and quantifies the SD between their FRET values. Larger SD than expected suggests changes in FRET during the burst.	Torella et al.^16^
Burst-wise fluorescence lifetime	E-τ	By comparing FRET efficiency with the donor fluorescence lifetime, deviations from the expected linear relationship reveal dynamics.	Maus et al.^17^
Two-channel kernel-based density distribution estimator	FRET-2CDE	Analysis of the local density of acceptor photons around donor photons, and donor photons around acceptor photons, throughout the burst.	Tomov et al.^18^
Photon-by-photon hidden Markov modeling	H^2^MM	Maximum likelihood estimation based on analytical estimators for model parameters, derived using the Baum-Welch algorithm.	Pirchi et al.^19^
Time-resolved burst variance analysis	trBVA	Expands BVA to provide quantitative kinetic rates.	Terterov et al.^20^

References were chosen to reflect a technique-specific application to the analysis of dynamics in diffusion-based smFRET. Please note that recurrence analysis of single particles (RASP),^21^ correlation-based approaches,^22^ photon distribution analysis (PDA),^23^^,^^24^ and Monte Carlo diffusion-enhanced photon inference (MC-DEPI)^25^ are omitted as they do not provide an individual burst-level classification of dynamic events.

## Results

### Benchmarking smFRET dynamic analysis methods

#### Sensitivity across kinetic regimes

To benchmark the performance of various dynamic analysis methods, smFRET data were simulated using PyBroMo^26^^,^^27^^,^^28^ (see STAR Methods). Here, “dynamic analysis” refers to the detection and quantification of transitions between distinct FRET efficiency states during the observation of individual molecules. Data were simulated to represent transitions between two distinct FRET efficiency states of E_1_ = 0.20 and E_2_ = 0.80 and a K = 1. These simulations were conducted across a range of kinetic regimes, defined by observed rate constants (k_obs(sim)_) of 1, 10^3^ and 10^6^ s^−1^ (corresponding to dwell times in each FRET state of 2 s, 2 ms, and 2 μs, respectively). Simulation parameters were derived from 15 experimental configurations reported in a multi-laboratory benchmarking study that determined the FRET efficiencies of several dye-labeled DNA duplexes obtaining FRET efficiencies with a standard deviation < 0.05^7^, ensuring that these simulated data closely mimic the characteristics of real-world measurements (see STAR Methods). This framework enabled a controlled evaluation of each method’s sensitivity to dynamic exchange under well-defined conditions. For all the methods of dynamic analysis used in this manuscript, both for simulated data and data collected in the laboratory, experiments are performed under the same regime, meaning that all these methods of analysis can be applied to the same raw data.

**Figure 2:**
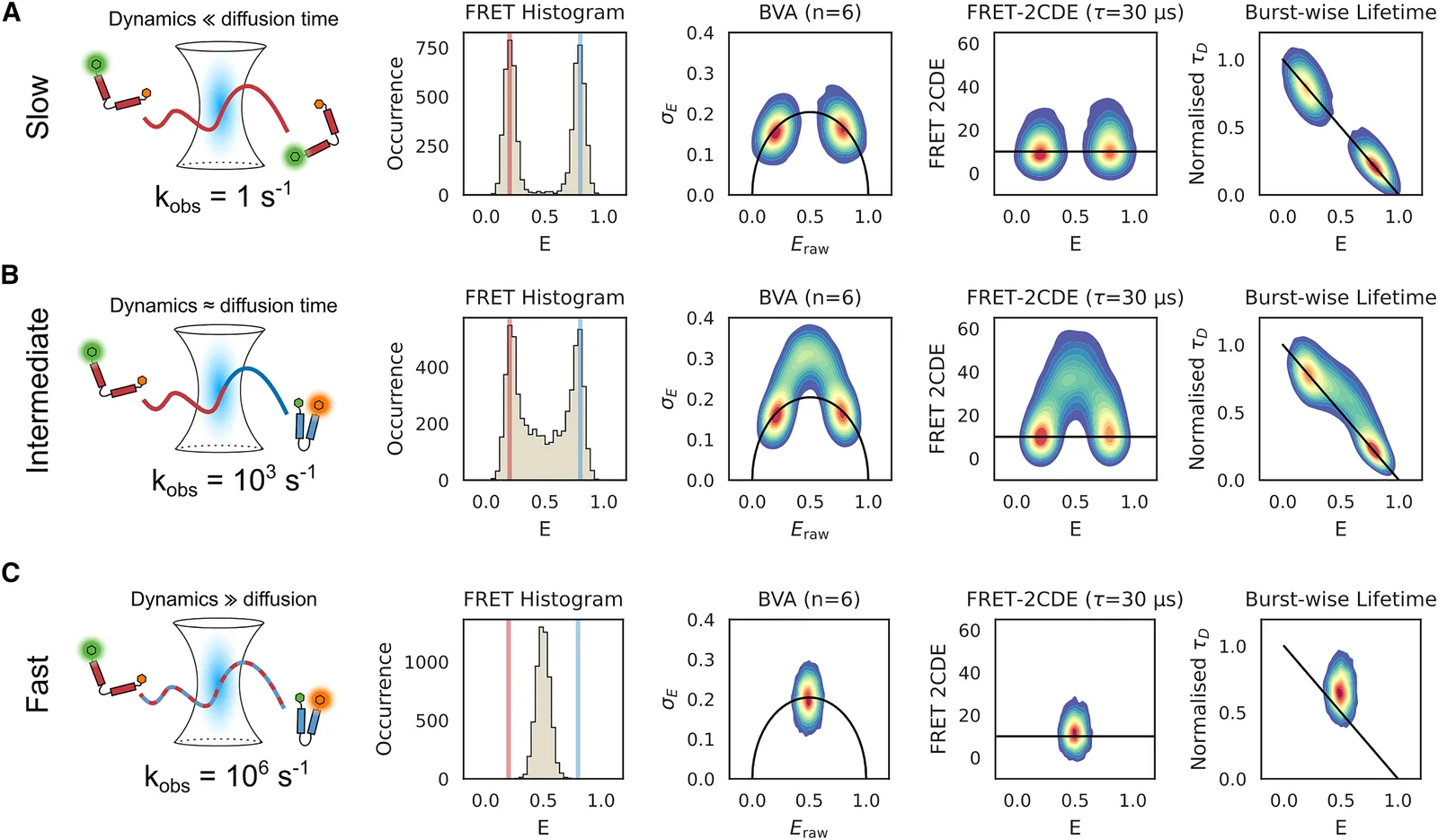
Effect of exchange kinetics on the readouts of smFRET dynamic analysis methods Simulated bursts from a two-state system (E1 = 0.20, E2 = 0.80; ΔE = 0.60; K = 1) are shown for three observed rate constants. Bursts were selected using a minimum threshold of 6 times the background rate and a minimum burst size of 60 photons, in line with the rest of the analysis (see STAR Methods). Each row shows, from left to right, an illustrative confocal volume with an example molecule diffusing through (colored line) converting between a low-FRET state (red) and a high-FRET state (blue) (not to scale), FRET histogram with vertical lines indicating the ground-truth FRET values of each state, burst variance analysis (BVA; *n* = 6), FRET two-channel kernel-based density distribution estimator (FRET-2CDE; τ = 30 μs), and burst-wise fluorescence lifetime analysis. Solid black lines represent the expected relationship between each analysis parameter and E for molecules that do not change FRET state during the burst (the static line); density above this line indicates dynamics during the burst. For an explanation of the parameters used in each analysis and illustrative examples of static and dynamic cases, see Figure S1. (A) Slow exchange relative to the diffusion time (k_obs(sim)_ = 1 s^−1^). (B) Intermediate exchange relative to the diffusion time (k_obs(sim)_ = 10^3^ s^−1^). (C) Fast exchange relative to the diffusion time (k_obs(sim)_ = 10^6^ s^−1^).

Inspection of FRET histograms can provide insight into conformational dynamics when the underlying kinetic model is known. For example, in “slow” exchange regimes where k_obs_ ≪ 1/diffusion time (τ_diff_), distinct separation between FRET populations is readily discernible (Figure 2A; FRET histogram). Here, mean τ_diff_ ≈ 2 ms after burst selection (Figure S2A). This increase in mean burst duration is expected because, for a fixed molecular brightness, longer bursts correspond to larger burst sizes (more detected photons) and the selection criteria bias the dataset toward these events. Under these conditions, the probability of state interconversion during the typical observation window is low, thereby preserving population integrity within the histogram. It is important to emphasize that clear separation between FRET populations should not be interpreted as evidence for the absence of underlying conformational dynamics. Instead, this observation can also indicate that the timescale of transitions is substantially slower than the diffusion-limited observation time, a distinction that is frequently not made. BVA, FRET-2CDE, or E-τ all show no clear dynamic signal, and density resides on the solid black lines, which represent the expected behavior for no dynamics on the diffusion timescale (Figure 2A; BVA, FRET-2CDE, and burst-wise fluorescence lifetime). An example of a purely static simulation with FRET values ranging between 0 and 1 is shown in Figure S2B. For a detailed explanation of the parameters used in each analysis method and illustrative examples of static and dynamic cases, see Figure S1.

When k_obs_ ≈ 1/τ_diff_, clear merging of the FRET states is seen, with some molecules interconverting during the burst and some not, creating what appears to be a “bridge” between the two FRET populations (Figure 2B; FRET histogram). At this timescale, all techniques tested performed well to observe dynamics in the simulated data, with the peaks remaining on the solid black lines (static during the burst) and the dynamically averaged intermediates rising above the black line, indicating dynamics during the burst (Figure 2B; BVA, FRET-2CDE, and burst-wise fluorescence lifetime).

In “fast” exchange regimes, where k_obs_ ≫ 1/τ_diff_, FRET histograms exhibit a single, dynamically averaged population (Figure 2C; FRET histogram). In such cases, methods that rely on the temporal distribution of photons within bursts are constrained in their ability to resolve dynamics due to the rate of exchange being much faster than the experimental mean inter-photon rate (≈10^5^ s^−1^). Consequently, methods that rely on the photon arrival time distribution within bursts struggle to resolve the underlying dynamics, as can be seen in BVA and FRET-2CDE residing on the “static” black line and the burst-wise fluorescence lifetime remaining above (Figure 2C).

**Figure 3:**
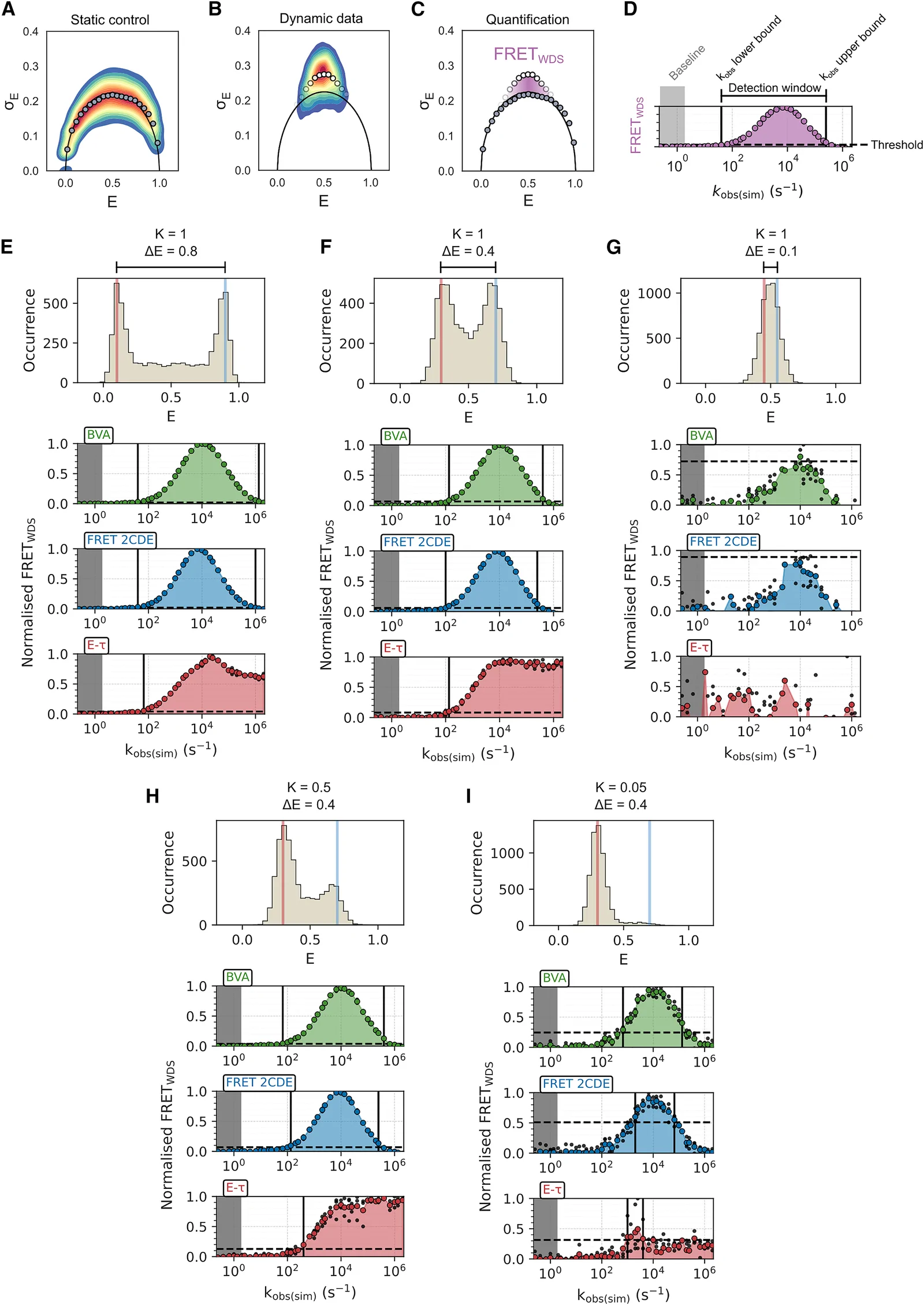
Sensitivity of smFRET dynamic analysis methods across exchange rates, FRET state separations, and equilibrium constants (A) Static control simulation with the mean σ_E_ value in each E bin shown as gray circles with a black outline. The expected static line is shown in black. (B) Dynamic sample (k_obs(sim)_ = 10^5^ s^−1^) showing deviation from the static line. Mean σ_E_ values in each E bin are shown as white circles with a black outline; marker transparency reflects the relative number of bursts in that bin. (C) Visual representation of how the FRET-weighted dynamic signal (FRET_WDS_; purple) is calculated as the difference between the observed signal and a static control (see STAR Methods). (D) Schematic of how the detection window is defined. The shaded gray region (k_obs(sim)_ ≤ 2 s^−1^) denotes the baseline assumed to appear static, from which the detection threshold (dashed horizontal black line) is calculated (see STAR Methods). Vertical black lines mark the lower (k_low_) and upper (k_high_) threshold crossings. (E–G) FRET histograms (top) and normalized FRET_WDS_ (bottom three rows) for a symmetric two-state system (K = 1) with ΔE values of 0.80 (E), 0.40 (F), and 0.10 (G). Vertical red and blue lines in the histograms indicate the ground-truth E values for the low- and high-FRET states, respectively. BVA (green; *n* = 6), FRET-2CDE (blue; τ = 30 μs), and E-τ (red) are shown across simulated k_obs_ values. Mean values are shown as colored circles; individual replicates are shown as black points. (H and I) As in (E)–(G) but for asymmetric equilibria with ΔE = 0.40: K = 0.5 (H), and K = 0.05 (I).

To assess systematically the sensitivity of different analysis methods across a wide dynamic range, k_obs_ was simulated spanning ∼10^−1^ to ∼10^6^ s^−1^ and the FRET weighted dynamic signal (FRET_WDS_; see STAR Methods) was derived as a measure of the dynamics within the data (Figures 3A–3D) for a range of different ΔE values (Figures 3E–3G) and a K = 1. For the low- and high-FRET combinations used to create the various ΔE values, see Table S1. Here, the two FRET states were centered around 0.50, which is where FRET is the most sensitive to changes in distance (Figure 1E). The ability to detect dynamics depends on how long each molecule is observed. For typical diffusion times (1–4 ms) the transition rate needed for 5% of bursts to contain a transition decreases only modestly, from 53 to 13 s^−1^ (Figure S3). This small shift makes this benchmarking broadly applicable, regardless of specific instrumentation or burst-selection settings.

For ΔE values of 0.80 and 0.40, this benchmarking revealed that BVA and FRET-2CDE begin to significantly underestimate dynamic contributions for k_obs(sim)_ > ∼10^4^ s^−1^, and for rates exceeding ∼10^5^ s^−1^, dynamic transitions become effectively masked, rendering the system indistinguishable from a static conformer (Figures 3E–3G; BVA and FRET-2CDE). In contrast, E-τ analysis leverages fluorescence lifetime information, thereby extending the temporal resolution limit beyond the inter-photon interval and instead constraining it by the donor fluorophore’s excited-state lifetime. This enables detection of substantially faster exchange kinetics (>∼10^5^ s^−1^) that would otherwise be inaccessible using traditional photon arrival time methodologies (Figures 3E–3G; E-τ). It is worth noting that the fluorescence lifetime of the acceptor following donor excitation is also sensitive to FRET, as the acceptor emission kinetics reflects a convolution of the donor and acceptor decay processes, and this information can similarly be used in E-τ analyses of dynamics.^29^^,^^30^ This study focuses on the donor lifetime implementation as it is the most widely adopted approach.

Experimental implementation of lifetime-resolved detection, however, requires time-correlated single-photon counting (TCSPC),^17^ which typically incurs significantly higher cost and complexity relative to standard confocal setups. Moreover, because pulsed excitation used for TCSPC can, in some dye systems, increase photobleaching relative to continuous-wave excitation at comparable average powers, achieving equivalent photon statistics may require longer acquisition times than with continuous-wave excitation.^31^ In the absence of lifetime information, systems undergoing rapid interconversion may misleadingly appear as a single static state, highlighting the necessity of matching analytical approaches to the kinetic regime of interest. For ΔE = 0.10, the range at which dynamic information can be deduced is reduced significantly for BVA and FRET-2CDE, and in the case of the burst-wise fluorescence lifetime analysis, it is not possible to distinguish at all. This is due to the small expected deviation from the static line^32^ (Figure S4A). For BVA, a segment size of *n* = 6 photons per segment was used (Figure S1A), corresponding to an effective time window centering around ∼19 μs (Figure S4B); larger segment sizes were not tested as they would reduce the number of windows per burst, potentially compromising the accuracy of the variance estimate. FRET-2CDE was evaluated across kernel widths (τ, μs; Figure S1C). Reducing τ enabled detection of faster exchange, whereas increasing this parameter did not extend the lower bound, presumably because it is limited by burst duration (Figure S4C).

The analysis presented above assumes symmetric exchange (K = 1), where both FRET states are equally populated. To assess how population asymmetry affects the detection of dynamics, additional simulations were performed across a range of equilibrium constants. Representative results for ΔE = 0.40 are shown for K = 0.5 (Figure 3H) and K = 0.05 (Figure 3I) which can be compared to K = 1 for the same ΔE in Figure 3F. At moderate asymmetry (K = 0.5), the FRET histogram shows visibly unequal population heights, but the detection performance of all three qualitative methods remains broadly comparable to the symmetric case (Figure 3F). At strong asymmetry (K = 0.05), where the minor state comprises only ∼5% of the population, the FRET histogram is dominated by a single peak, and the minor state is barely discernible. Despite this, BVA and FRET-2CDE still detect dynamics, albeit over a narrower rate window than in the symmetric case. This is notable given that only a small fraction of bursts samples the minor state. In contrast, E-τ is unable to resolve a meaningful dynamic signal under these conditions.

Dynamic processes in diffusion-based single-molecule FRET experiments are often contextualized with respect to the characteristic diffusion time of the molecule traversing the observation volume. While this reference point is informative, the analysis demonstrates that conformational dynamics can be reliably detected across a broad temporal range spanning at least 4 orders of magnitude (10^2^–10^6^ s^−1^) in the case of inter-photon methods, and up to the excited-state lifetime of the donor molecule (∼4 ns) in the case of the burst-wise lifetime analysis. Moreover, BVA and FRET-2CDE retain the ability to detect dynamics even when the minor state comprises only ∼5% of the equilibrium population, highlighting the sensitivity of smFRET analytical approaches and underscoring their utility in probing both slow and fast molecular transitions relative to the diffusion-limited observational window.

#### FRET separation, burst number, and equilibrium constants in reliable kinetic inference

**Figure 4:**
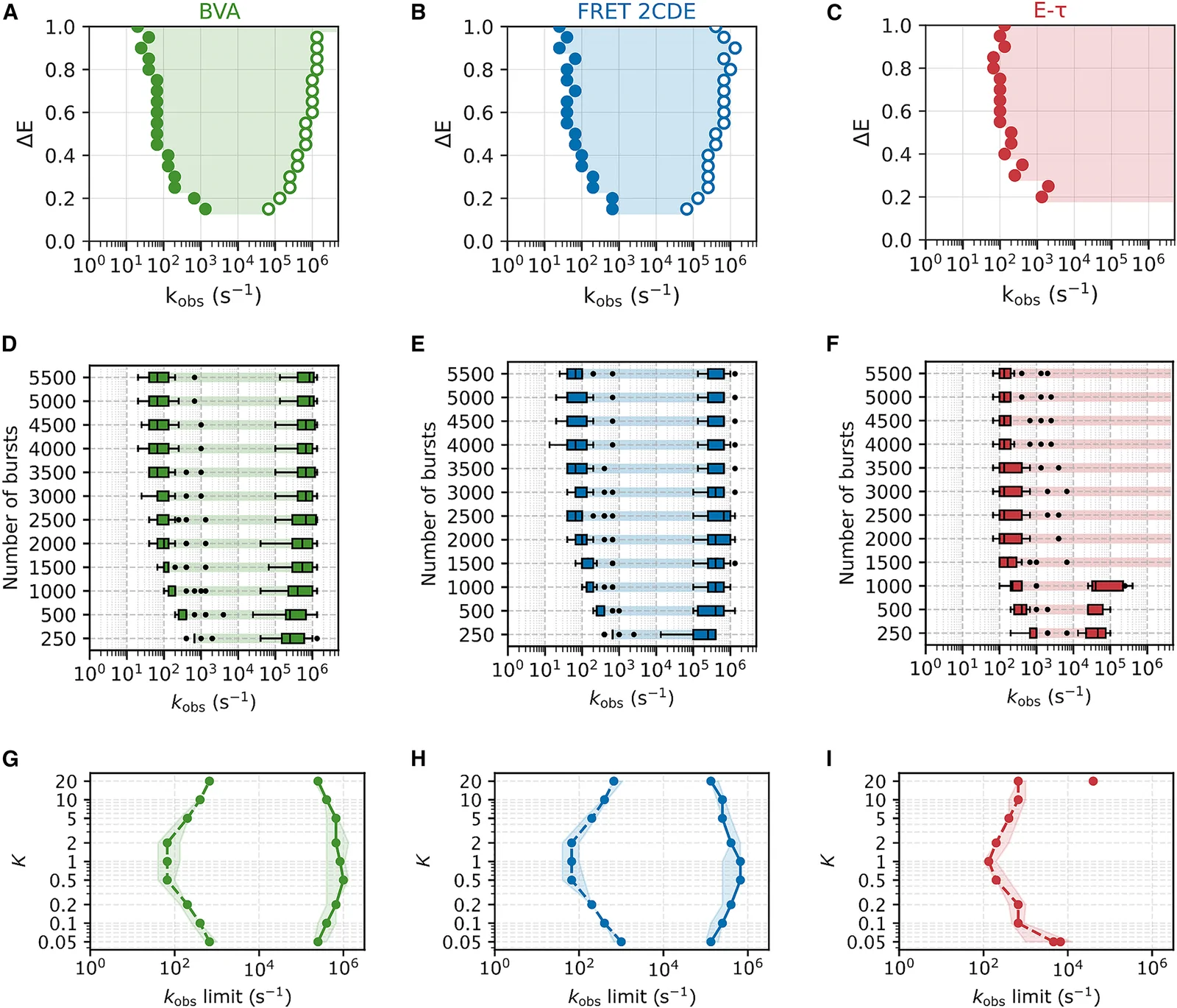
The detection limits of analysis methods are influenced by the FRET state separation (ΔE), burst count, and equilibrium constant (K) Columns show BVA (*n* = 6; left; green), FRET-2CDE (τ = 30 μs; center; blue), and burst-wise fluorescence lifetime (right; red). (A–C) Upper and lower bounds of k_obs_ as a function of ΔE for K = 1. Filled and hollow markers represent the lower and upper bounds, respectively. (D–F) Boxplots of the upper and lower bounds of k_obs_ as a function of the number of bursts in the analysis for ΔE ≥ 0.20 and K = 1. Boxes show the interquartile range (25th–75th percentile) with the median as a line; whiskers extend to the most extreme values within 1.5 times the interquartile range, and outliers are shown as black points. (G–I) Upper and lower bounds of k_obs_ as a function of K for ΔE ≥ 0.20. Dashed lines represent the lower bound, and solid lines represent the upper bounds of detection. Mean values are shown only where all three replicates exceeded the detection threshold; conditions not meeting this criterion are omitted to avoid misrepresentation by outliers.

To further probe the range of timescales accessible to different smFRET analysis methods, the lower (k_low_) and upper (k_high_) bounds of detected dynamics across various conditions were calculated (vertical lines in Figure 3D; see STAR Methods). To test the effect of FRET state separation on the detection of dynamics at different rates (∼10^−1^ to ∼10^6^ s^−1^), 20 different ΔE values between 1 and 0.05 were tested, centered around E = 0.50 (Table S1), across nine equilibrium constants (K = 0.05, 0.1, 0.2, 0.5, 1, 2, 5, 10, and 20). In the BVA and FRET-2CDE analysis for K = 1, detection of dynamics was possible for ΔE values as low as 0.15, but only over a limited range between k_low_ ≈ 10^3^ s^−1^ and k_high_ ≈ 4 10^4^ s^−1^ (Figures 4A and 4B). Inspection of the data from which the thresholds are derived shows that a broader detection range might be possible, but not with high confidence under these conditions (Figure 3G). Above a ΔE of 0.50, rates stayed consistent, spanning around 4 orders of magnitude from k_low_ ≈ 20 s^−1^ to k_high_ ≈ 5⋅ 10^5^ s^−1^ (Figures 4A and 4B). In contrast, the burst-wise fluorescence lifetime analysis struggled to detect dynamics when the states were separated by ΔE values smaller than 0.20 (Figure 4C). This outcome is not surprising considering the very small, expected deviation from the static line for states centered around E = 0.50 (Figure S4A). However, for multiple states centered around values higher or lower than 0.50, a larger deviation is expected, and detection of dynamics using burst-wise fluorescence lifetime analysis at smaller ΔE should be feasible.^32^ For ΔE values between 0.25 and 0.50, k_low_ decreases steadily from ∼10^3^ s^−1^ to ∼10^2^ s^−1^, remaining at ∼10^2^ s^−1^ at higher ΔE values (Figure 4C).

Another important factor is the total burst count. There is no consensus standard, as the optimal number depends on the application. In a recent multi-laboratory benchmarking study, the number of bursts per experiment ranged from ∼450 to ∼21,400.^8^ To evaluate how burst count influences the detection of dynamics, k_high_ and k_low_ were quantified as a function of the total burst number. Only FRET bursts are considered here. Real-world datasets often contain a substantial fraction of incompletely labeled biomolecules and may be affected by photo-blinking or photobleaching during the burst, all of which must be removed prior to analysis and comparison with the burst numbers seen here. To detect and remove bursts affected by photo-blinking or photobleaching, filters such as ALEX-2CDE^18^ and TGX-TRR^33^ can be employed. H^2^MM can also be used to identify blinking and bleaching events, which can then be excluded from the analysis.^34^^,^^35^ These filters are not mutually exclusive and can be applied in combination.

For BVA and FRET-2CDE, increasing the number of bursts used for analysis past 2,000 had little effect on k_high_ or k_low_ (Figures 4D and 4E). As expected, for the burst-wise fluorescence lifetime, generally no k_high_ is observed under the timescales simulated, except for in low burst counts (Figure 4F). For all methods tested, decreasing the burst count below 2,000 increased the k_low_, by an order of magnitude from ∼10^2^ s^−1^ to ∼10^3^ s^−1^, preventing slower dynamics from being detected (Figures 4D–4F). This trend is expected; as the transition events become more infrequent, a larger dataset is required to observe them. For the burst-wise fluorescence lifetime, some threshold crossings were detected for the k_high_ at low burst counts, where FRET_WDS_ is inherently noisier due to fewer data points contributing to the weighted average.

To assess how population asymmetry affects the detection windows, k_low_ and k_high_ were examined as a function of K for ΔE ≥ 0.20 (Figures 4G–4I). For BVA and FRET-2CDE, the detection windows remained broadly stable across moderate asymmetries (K = 0.2 to 5), with both k_low_ and k_high_ comparable to the symmetric case (Figures 4G and 4H). At asymmetries beyond this range (K ≤ 0.2 or K ≥ 5), k_low_ increased and k_high_ decreased, narrowing the accessible rate window from approximately four orders of magnitude (∼10^2^ s^−1^ to ∼10^6^ s^−1^) to two (∼10^3^ s^−1^ to ∼10^5^ s^−1^). The burst-wise fluorescence lifetime analysis showed a directional sensitivity to population bias (Figure 4I). For increasing K, where the high FRET state becomes the major population, the detection window followed trends similar to BVA and FRET-2CDE. For decreasing K, however, sensitivity to dynamics was reduced more substantially. This likely reflects the fact that at low K values, the high FRET state is the minor population, and because the burst-wise donor lifetime is a photon-weighted average, transient visits to a high FRET state contribute disproportionately few donor photons, diminishing their influence on the mean lifetime and reducing the deviation from the static line.

Overall, these results demonstrate that with sufficient burst counts and well-separated FRET states, smFRET can quantify dynamics across an impressive kinetic range spanning up to four orders of magnitude with BVA or FRET-2CDE and more using burst-wise fluorescence lifetime (approximately six orders of magnitude). However, as ΔE decreases, the accessible range narrows substantially, lower burst counts disproportionately affect the range of timescales accessible, and population asymmetry further compresses the detection window, particularly for the burst-wise fluorescence lifetime at low K values. Together, these factors highlight the need to use caution when interpreting data from closely spaced FRET states, limited datasets, or systems with strongly unequal state populations.

#### Quantitative extraction of interconversion rates

To move from simply detecting dynamics to quantifying them, this section evaluates how accurately the underlying interconversion rates between FRET states can be recovered, focusing on photon-by-photon hidden Markov modeling (H^2^MM). H^2^MM was originally developed by Pirchi et al.^19^ to apply HMM to photon arrival times, which are the primary readout in diffusion-based smFRET experiments. This technique was later extended by Harris et al. (mpH^2^MM) to incorporate stoichiometry (additional information gained from alternate excitation regimes^36^) and fluorescence lifetime information^37^^,^^38^ to better define both the correct number of FRET states and photo-blinking/bleaching than just H^2^MM alone. Although time-resolved burst variance analysis (trBVA) (Table 1) also provides quantitative rate extraction, no open-source implementation was available at the time of writing, limiting its accessibility to the broader community. In this manuscript, H^2^MM is used for rate extraction for four main reasons: (1) it has an actively maintained open-source implementation (burstH2MM); (2) it is quickly gaining traction in the field^34^^,^^39^^,^^40^^,^^41^^,^^42^^,^^43^^,^^44^^,^^45^^,^^46^; (3) it can directly remove bursts containing fluorophore photo-blinking, which has been shown to influence derived rates^35^; and (4) although not examined here, it is capable of deriving rate constants for transitions between more than two FRET states.^37^ For a comprehensive overview of the techniques for the quantitative analysis of diffusion-based smFRET data, the reader is referred to the recent review by Gopich and Chung.^47^ In this section, it is assumed that the correct number of states has already been determined, which can either be achieved through the H^2^MM process itself or through techniques such as sub-ensemble fluorescence lifetime fitting.^48^

**Figure 5:**
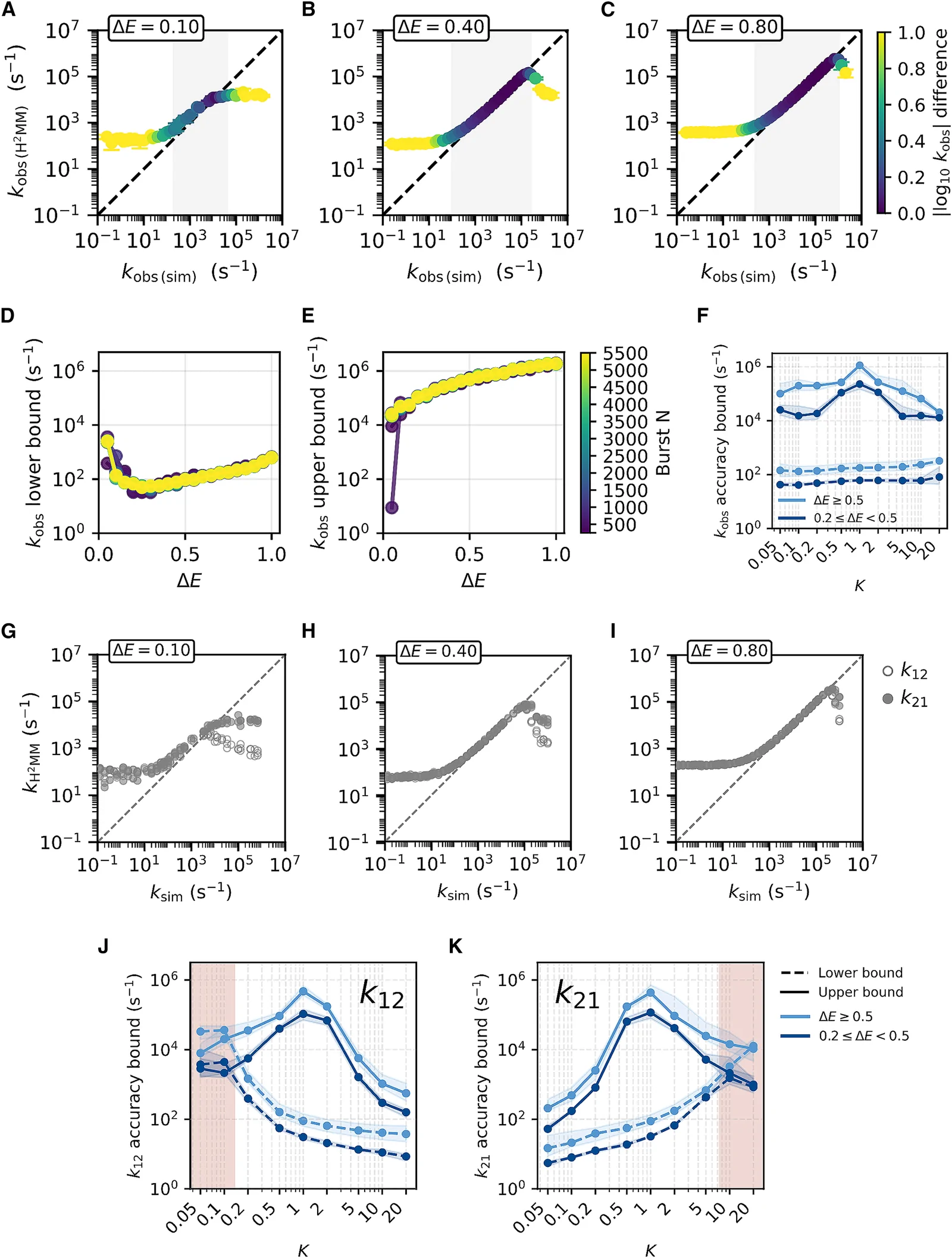
Accuracy and dynamic range of H^2^MM-derived rate estimates across different ΔE values, burst numbers, and equilibrium constants (A–C) Comparison of H^2^MM-estimated k_obs_ values (k_obs(H2MM)_) with the ground truth of the simulation (k_obs(sim)_) for (A) ΔE = 0.10, (B) ΔE = 0.40, and (C) ΔE = 0.80. Points are colored by the absolute log_10_ difference between estimated and simulated rates, with gray shaded regions indicating the range of k_obs_ values meeting an agreement threshold |log_10_(k_obs(H2MM)_/k_obs(sim)_)| ≤ 0.5. The black dashed diagonal line represents perfect agreement. Error bars represent one standard deviation of three replicates. (D–F) Dependence of the (D) lower and (E) upper accuracy bounds on ΔE for different total burst numbers (burst N; ranging from 250 to 5,500). (F) Accuracy bounds of k_obs_ as a function of the equilibrium constant (K) for ΔE ≥ 0.50 (light blue) and 0.20 ≤ ΔE < 0.50 (dark blue); dashed and solid lines represent the lower and upper bounds, respectively. (G–I) Comparison of individual H^2^MM-estimated rate constants k_12_ (open circles) and k_21_ (filled circles) with the corresponding simulated rates (k_sim_) for (G) ΔE = 0.10, (H) ΔE = 0.40, and (I) ΔE = 0.80. The gray dashed diagonal line represents perfect agreement. (J and K) Accuracy bounds for (J) k_12_ and (K) k_21_ as a function of K for ΔE ≥ 0.50 (light blue) and 0.20 ≤ ΔE < 0.50 (dark blue); dashed and solid lines represent the lower and upper bounds, respectively. Red shaded regions indicate conditions where accurate rate extraction is not possible. Data in (A)–(C) and (G)–(I) are shown for 5,500 bursts with K = 1. Data in (F), (J), and (K) are shown for 5,500 bursts.

The dependence of the accuracy of the H^2^MM model was evaluated against the separation of FRET states (Figures 5A–5C). In all cases, k_obs(sim)_ diverges from k_obs(H2MM)_ at rates < ∼200 s^−1^, around the diffusion timescale, a trend also reported by others.^35^^,^^38^ Larger FRET state separation allowed for accurate determination of rates at faster timescales: for example, ΔE = 0.10 allowed extraction of rates within 0.5 log_10_ difference of a k_obs_ of ∼5 10^4^ s^−1^ (Figure 5A), while increasing the ΔE to 0.80 extended this upper bound by over an order of magnitude to ∼10^6^ s^−1^ (Figure 5C). Considering all simulated ΔE values (Table S1), a clear relationship emerges between ΔE and the lower and upper bounds of k_obs_ (Figures 5D and 5E). Notably, varying the number of bursts in the analysis did not affect the lower (∼10^2^ to ∼10^3^, Figure 5D) or upper bounds (∼10^4^ to ∼10^6^, Figure 5E) of k_obs_. The equilibrium constant also influenced the accessible dynamic range (Figure 5F): while the lower bound remained largely unchanged across K values, the upper bound decreased as K deviated from unity, falling from ∼10^6^ s^−1^ at K = 1 to ∼10^5^ s^−1^ for larger ΔE and ∼10^4^ s^−1^ for smaller ΔE at more extreme K values.

Beyond k_obs_, the ability of H^2^MM to recover the individual rate constants k_12_ and k_21_ was assessed (Figures 5G–5I). At K = 1, where k_12_ and k_21_ are equal, the ratio between the two rates is well preserved up to ∼10^3^ s^−1^, above which the estimates diverge from one another (Figure 5G). At higher ΔE values, H^2^MM maintains this balance more effectively across a wider range of rates (Figures 5H and 5I), consistent with the improved k_obs_ recovery observed in Figures 5A–5C. The effect of K on the extraction of individual rates was more pronounced than for k_obs_ (Figures 5J and 5K). At extreme K values, the rate constant associated with the sparsely populated state becomes unrecoverable: k_12_ cannot be reliably determined at low K, where the high-FRET state is minimally occupied (Figure 5J), while k_21_ fails under the opposite conditions (Figure 5K). Increasing the number of bursts in the analysis may help mitigate this in extreme cases by improving sampling of the minor population. These results highlight that extracting individual rate constants is considerably more challenging than recovering k_obs_ and that the equilibrium constant strongly shapes which rates are accessible. Researchers using H^2^MM to characterize the conformational landscape of proteins should, therefore, exercise caution, particularly when working with systems that exhibit heavily skewed equilibria.

In addition to rate extraction, the accuracy of the FRET state efficiencies returned by H^2^MM was assessed (Figure S6). At K = 1, retrieved efficiencies closely matched the ground truth across all kinetic regimes and ΔE values, with only modest increases in variability at low burst counts. However, at extreme K values, retrieval of the minor-state efficiency became substantially less reliable, particularly in the fast exchange regime and at low ΔE, mirroring the limitations observed for individual rate constant extraction.

This analysis provides an opportunity to benchmark the ability of H^2^MM to correctly determine the number of FRET states in our simulated data across a broad range of k_obs_, ΔE, and K values. To assess the model selection accuracy, integrated completed likelihood (ICL)^37^^,^^49^^,^^50^ and the modified Bayesian information criterion (BIC′)^51^ were applied (Figure S1D), and the percentage of correct assignments for different k_obs_ ranges with different ΔE values (Figure S7A) and K values (Figure S7B) was calculated. The results show that the better-performing criterion depends strongly on the kinetic regime: ICL is preferable for well-separated states at slow and intermediate timescales, whereas BIC′ is more reliable at fast timescales (k_obs_ ≥ 10^5^ s^−1^) and for moderately separated states once kinetics exceeds 10^2^ s^−1^. Accuracy is largely insensitive to K, and both methods show little improvement above ∼1,000 bursts, matching what was observed for the rate bounds (Figures 5D and 5E). Figure S7 is intended as a reference to guide which criterion to favor when the two disagree.

Taken together, these results demonstrate that while H^2^MM can reliably extract k_obs_ across a broad dynamic range, derived rates should be interpreted with care. Individual rate constants k_12_ and k_21_ are subject to additional limitations imposed by ΔE, K, and the accessible timescale and should not be treated as quantitatively reliable without first considering these factors.

## Discussion

**Figure 6:**
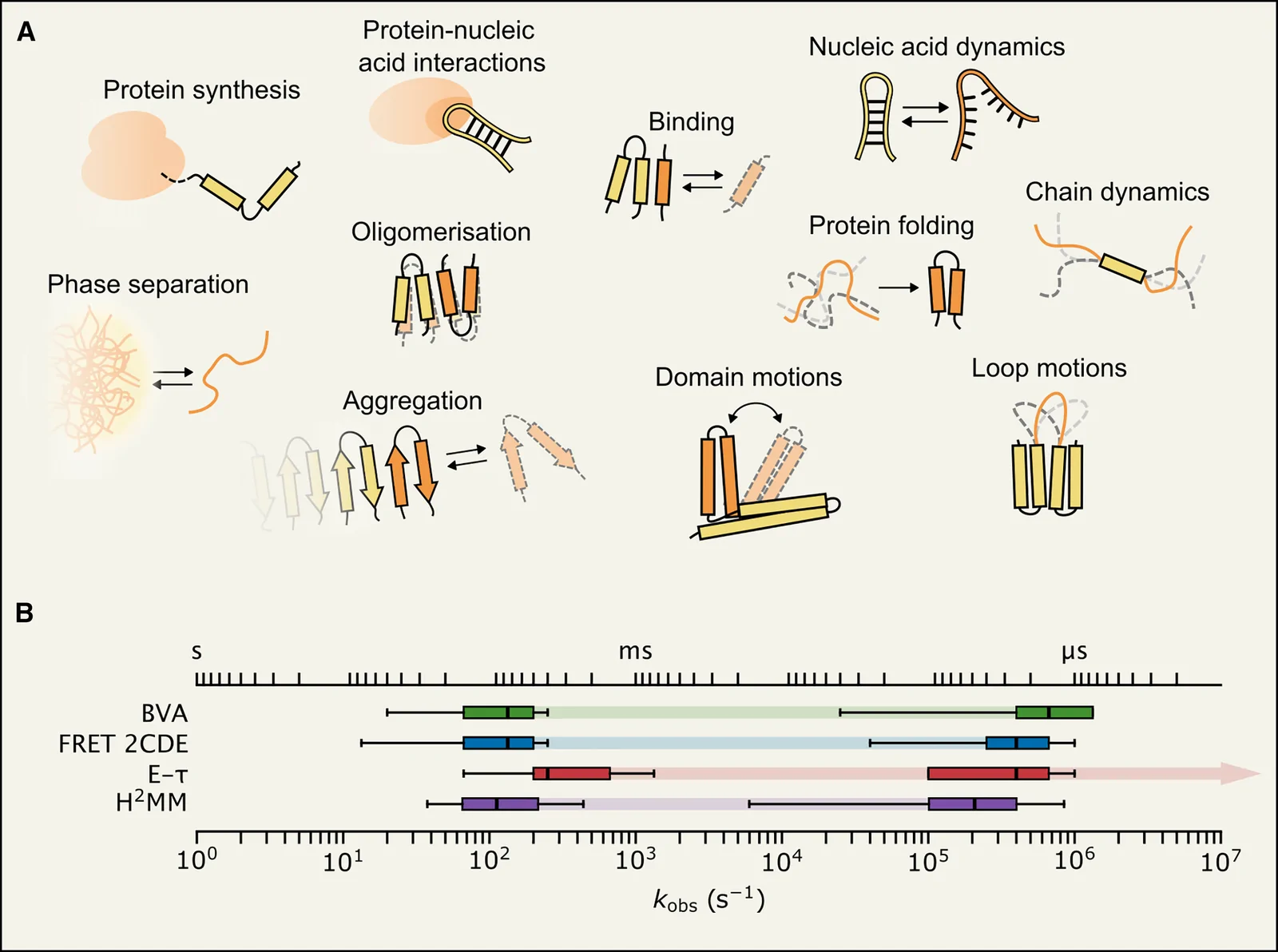
Detecting biomolecular dynamics across a wide kinetic range using diffusion-based single-molecule FRET (A) Examples of biomolecular processes accessible to single-molecule FRET experiments, spanning diverse structural scales and kinetic regimes. (B) Detection limit distributions for BVA, FRET-2CDE, E-τ, and H^2^MM (accurate rate retrieval range) across simulated FRET pairs with ΔE ≥ 0.20, at a burst count of 5,500 and pooled across equilibrium constants K = 0.2–5. Horizontal lines span the range over which each method can reliably detect transitions (i.e., between k_low_ and k_high_). Boxes show the interquartile range (25th–75th percentile) with the median as a line; whiskers extend to the most extreme values within 1.5 times the interquartile range, outliers are not shown.

This manuscript presents a quantitative, simulation-backed dataset that directly links diffusion-based smFRET dynamic analysis methods to the kinetic regimes they can reliably report on for a two-state FRET system (Figure 6A). Inter-photon methods such as BVA and FRET-2CDE can readily detect observed exchange rates between ∼30 and ∼10^6^ s^−1^, with K = 0.2–5; at K = 1 specifically, this corresponds to dwell times in each FRET state of ∼67 ms and ∼2 μs. Burst-wise fluorescence lifetime analysis (E-τ) extended this range to include rates >10^6^ s^−1^ making the latter uniquely suited for ultra-fast exchange, as is found in unfolded proteins or intrinsically disordered proteins,^52^^,^^53^ albeit at the cost of more complex and expensive hardware. H^2^MM yields quantitative k_obs_ estimates within a ΔE-dependent window spanning up to four orders of magnitude (∼10^2^ s^−1^ to ∼10^6^ s^−1^), although extraction of the individual rate constants k_12_ and k_21_ is further constrained by the equilibrium constant, with rates associated with sparsely populated states becoming unreliable at extreme K values. Understanding FRET states, their populations, and the transition rates between them enables the reconstruction of conceptual energy landscapes that illustrate and help interpret potential functional and dysfunctional mechanisms.^34^^,^^54^^,^^55^

Together, these results lay out a general rule set for accessible timescales (Figure 6B) and introduce a practical benchmarking framework that researchers can adapt to specific biological contexts and their own experimental conditions, including parameters such as diffusion time, signal-to-noise ratio, number and separation of FRET states, and dataset size. A subset of the simulated data, representative of a typical smFRET experimental setup, is released alongside the code to reproduce the full data or generate custom datasets, providing a resource for both optimizing existing workflows and developing new analysis methods.

The benchmarking presented here highlights not only the importance of matching analysis methods to the kinetic regime of interest but also the capability of diffusion-based smFRET to access an exceptionally broad temporal range under native-like conditions. Few techniques can probe dynamics from tens of milliseconds to sub-microseconds in freely diffusing molecules, at room temperature, and at picomolar concentrations. This breadth is particularly valuable as it can be achieved without surface tethering, enabling experiments in complex and crowded environments such as liquid-liquid phase separation^56^^,^^57^^,^^58^ and even within living cells.^59^^,^^60^ These results provide a framework for leveraging smFRET’s unique ability to link molecular structure and dynamics across broad timescales in biologically relevant environments.

Beyond the kinetic windows benchmarked here, sensitivity can be extended through experimental formats and analysis strategies that improve trajectory length and photon statistics. The anti-Brownian electrokinetic (ABEL) trap and confocal measurements of surface-tethered molecules both generate much longer trajectories than diffusion-limited bursts,^61^ increasing sensitivity to slower dynamics while retaining the fast-dynamics advantage of single-photon detection, although tethering comes with possible surface perturbations. Zero-mode waveguides^62^ provide a complementary route to faster dynamics by enabling substantially higher detected photon rates and improved short-timescale photon statistics, potentially increasing sensitivity to rapid transitions by decreasing the mean inter-photon time.^63^ In parallel, deep-learning-based analysis represents an attractive future direction for diffusion-based smFRET, with potential to improve denoising, state assignment, and rate inference in low-photon, heterogeneous, or weakly separated systems, and thereby extend practical detection limits beyond current workflows. Together, these approaches provide clear routes for pushing beyond the ranges identified here, while the benchmarking framework introduced in this study offers a basis for quantifying such gains under matched conditions.

A practical guide for analysis method selection, based on the benchmarking results presented above, is provided in Table S2. The starting point for method selection is the appearance of the FRET efficiency histogram, which provides an initial indication of the likely kinetic regime. Clearly separated FRET populations should not be interpreted as confirmation that the system is static, as exchange slower than the diffusion-limited observation window is indistinguishable from a truly static system by histogram inspection alone. Equally, a single averaged population does not imply structural homogeneity, as fast exchange above the inter-photon rate produces the same appearance in the absence of lifetime-resolved detection. The boundaries between regimes are approximate and condition dependent; quantitative thresholds tailored to specific experimental parameters are available in the accompanying simulated datasets.

Analysis method selection is best treated as an explicit, system-matched design choice, not merely a question of whether dynamics are present within a sample. The benchmarking and datasets provided alongside this study offer a practical starting point for making these choices with influence from such factors as burst counts, ΔE, and expected k_obs_. Adoption of such an approach ensures that interpretation of smFRET data is governed by the underlying dynamics, not methodological constraints.

### Limitations of the study

All simulations used a single set of photon emission and background rates, derived from the multi-laboratory mean reported by Agam et al.^8^ Detection limits will differ under other experimental conditions, particularly with lower molecular brightness or higher background. Researchers are encouraged to simulate their own parameters using the provided framework to establish instrument-specific detection limits.

The simulations are restricted to two-state systems. Extending to three or more states would scale the parameter space by orders of magnitude, beyond what is tractable within a single study. Moreover, multi-state analysis first requires determining the number of states present, which remains a challenge. Benchmarking multi-state detection and rate extraction is an important direction for future work.

All FRET state pairs were centered around E = 0.50, where burst-wise fluorescence lifetime sensitivity to dynamics is at its lowest. States centered at higher or lower E values would produce larger deviations from the static line, so the detection limits reported here for this method should be considered conservative.

## Resource availability

### Lead contact

Requests for further information and resources should be directed to and will be fulfilled by the lead contact, Joel A. Crossley (j.a.crossley@leeds.ac.uk).

### Materials availability

This study did not generate new unique reagents.

### Data and code availability

• The full simulated dataset is approximately 1.5 TB and is not deposited in its entirety. A representative subset of the simulated smFRET data and the complete set of analysis output files (CSV) underlying every figure containing the full data has been deposited at Zenodo (https://doi.org/10.5281/zenodo.19352623).
• All original code used to generate the simulations and to reproduce the results reported in this study has been deposited at Zenodo (https://doi.org/10.5281/zenodo.19352623).
• Any additional information required to re-analyze the data reported in this study is available from the lead contact upon request.

## Acknowledgments

The author acknowledges the support of the 10.13039/501100000777University of Leeds and 10.13039/501100000268BBSRC (BB/Y00034X/1). Gratitude is extended to all members of the Sheena E. Radford, David J. Brockwell, and Antonio N. Calabrese labs for insightful discussions and support and to Paul D. Harris for insightful discussions into the H^2^MM analysis. Appreciation is extended to Luke A. Johnson for providing helpful feedback on the figures. This work was undertaken on the Aire HPC system at the 10.13039/501100000777University of Leeds, UK. Appreciation is expressed to the community of researchers whose open-source tools and software provided the foundation for the simulations and analysis presented in this manuscript.

## Author contributions

J.A.C. conceptualization, data curation, formal analysis, investigation, methodology, software, visualization, writing – original draft, and writing – review and editing.

## Declaration of interests

The author declares no competing interests.

## Declaration of generative AI and AI-assisted technologies in the writing process

During the preparation of this work, the author used Claude by Anthropic for manuscript proofreading. After using this tool, the author reviewed and edited the content as needed and takes full responsibility for the content of the publication.

## STAR★Methods

### Key resources table

**Table:** 

REAGENT or RESOURCE	SOURCE	IDENTIFIER
**Deposited data**		

Representative Dataset	This study	https://doi.org/10.5281/zenodo.19352623

**Software and algorithms**		

Code Repository	This study	https://doi.org/10.5281/zenodo.19352623
Python (3.10)	Python Software Foundation	https://www.python.org/
FRETBursts (0.8.3)	Ingargiola et al.^64^ OpenSMFS	https://github.com/OpenSMFS/FRETBursts
PyBroMo (0.8.1)	OpenSMFS	https://github.com/OpenSMFS/PyBroMo
phconvert (0.9.1)	Ingargiola et al.^65^ Photon-HDF5	https://github.com/Photon-HDF5/phconvert
burstH2MM (0.1.7)	Harris et al.^37^	https://github.com/harripd/burstH2MM
Burst variance analysis	Torella et al.^16^	https://doi.org/10.1016/j.bpj.2011.01.066
Two-channel kernel-based density distribution estimator	Tomov et al.^18^	https://doi.org/10.1016/j.bpj.2011.11.4025
Photon-by-photon hidden Markov modeling	Pirchi et al.^19^	https://doi.org/10.1021/acs.jpcb.6b10726

**Other**		

High performance computing facility (Aire)	University of Leeds	N/A

### Method details

#### Data simulation

smFRET data were simulated using the open-source PyBroMo Python package (Version 0.8.1).^26^ In short, the 3D Brownian motion of particles is simulated in a box, the photon emission profile for each particle is then calculated based on an assigned FRET value and their maximum emission rate according to their position in relation to a modeled excitation point spread function (PSF). These photon trajectories are then saved to Photon-HDF5 file format. In the case of simulating dynamics, two photon trajectories with different FRET values were simulated from the same Brownian motion simulation before being combined in an alternating fashion according to the rate of interchange which is being simulated (see below for more detail).

The PSF used to describe the confocal volume was the default numeric model in PyBroMo, which had been modeled using PSFLab for a water immersion objective (NA = 1.2) at 532 nm, accounting for effects like refractive index mismatch and discrepancies between the objective lens correction and cover glass thickness. The PSF was centered in a box with dimensions of x = 8.0 μm, y = 8.0 μm and z = 9.2 μm. The box contained 10 molecules, to give a molar concentration of ∼30 pM. This concentration was chosen to minimize the probability of multiple molecules simultaneously occupying the detection volume, which has been shown to introduce impure bursts that systematically bias retrieved FRET efficiencies.^27^ A diffusion coefficient (D) of 9.18 10^−11^ m^2^/s was used as calculated for a spherical particle with a radius (d, in meters) of 2.4 nm, which equates to a globular protein of ∼50 kDa, using the equation

$$D=\frac{k_{B}\cdot T}{6\cdot \pi \cdot \eta \cdot d}$$

where k_B_ is the Boltzmann constant (1.38 10^−23^ J/K), T is the temperature (294.15 K; 21°C) and *η* is the viscosity of water at 294.15 K (9.778 10^−4^ Pa s). Brownian motion simulations had a step size of 0.5 μs and a length of 3600 s. While the mean inter-photon time across a burst remains longer than the simulation step size, at the fastest simulated rates the mean dwell time in each state begins to approach it; results at these extremes should therefore be interpreted with some caution, and researchers wishing to simulate dynamics at comparable or faster timescales should be mindful of this caveat when selecting an appropriate step size.

To generate photon timestamp data from the particle simulation, photon emission rates and background rates were set to values equal to the averages from 16 different labs reported by Agam et al*.*^8^ A peak emission rate for the sum of the donor and acceptor was set to 271.55 kHz. Please note that the peak emission rate refers to the maximum rate at the center of the PSF, not the average rate experienced throughout a burst; molecules traversing the confocal volume spend the majority of their transit time away from the PSF center, so the mean detected photon rate per burst is substantially lower than this figure (see Figure S5 for examples of the average rates). A background rate of 1.414 and 0.616 kHz was set for the donor and acceptor channels respectively. Photon nanotimes, fluorescence lifetime states and instrument response function were added to the simulation using the methods derived by Harris et al*.*^38^ The fluorescence lifetime assigned to the donor photon stream in each state (τ_state(i)_) was calculated with the following equation:

$$\tau_{\text{state}\left(i\right)}=\tau_{\max}\cdot \left(1-E_{\text{state}\left(i\right)}\right)$$

where τ_max_ is the ‘maximum’ lifetime, equivalent physically to the donor in the absence of an acceptor molecule, (set here to 4.1 ns; equivalent to the commonly used donor dye Alexa Fluor 488) and E_state(i)_ is the FRET value in the state. The acceptor photon stream was given a lifetime of 2.8 ns (with no change during FRET), although it was not used for any form of analysis in this work. This was done to generate 21 data files, for FRET states between 0 and 1. These data files were then mixed according to a transition rate (k_obs(sim)_) to generate files with dynamic interchange between two FRET states. Data files were mixed to generate 20 ΔE values between 0.05 and 1 (i.e., in 0.05 E steps; Table S1) and 42 transition rates for each of those (∼log spaced values between 0.125 s^−1^ and 1,000,000 s^−1^ for k_12_ and k_21_; equivalent dwell times of 8 s and 1 μs in each state).

The initial Brownian motion simulation and the subsequent photon trajectory simulation were done in triplicate with different initial starting seeds (seeds = 1, 2, and 3), to produce independent replicates. Static FRET control simulation was generated by combining 21 photon emission simulations, each derived from the same Brownian motion simulation used for the dynamic cases, with E values incrementing from 0 to 1 in steps of 0.05. The maximum photon emission rates, background and fluorescence lifetime values were set the same as for the dynamic cases. The simulations described above used symmetric exchange (K = 1), where k_12_ = k_21_ = k_obs_/2. To examine the effect of asymmetric exchange, additional simulations were performed for eight further equilibrium constants (K = 20, 10, 5, 2, 0.5, 0.2, 0.1 and 0.05), where K = k_12_/k_21_. For each value of K, the same 42 k_obs_ values and 20 ΔE conditions were used, with the individual forward and reverse rate constants calculated as k_12_ = k_obs_ ⋅ K/(1 + K) and k_21_ = k_obs_/(1 + K). Combined with the original K = 1 condition, this yields nine equilibrium conditions and a total of 22,680 one-hour Photon-HDF5 files for subsequent smFRET analysis. The number of bursts detected per file was a function of the burst selection criteria (a background multiplier of 6 and a minimum burst size of 60 photons), yielding approximately 5,800 bursts per file and approximately 130 million bursts in total. Each condition was also tested across 12 burst counts (250, 500, 1,000, 1,500, 2,000, 2,500, 3,000, 3,500, 4,000, 4,500, 5,000 and 5,500), resulting in a total of 272,160 analysis runs.

#### Data analysis

##### Burst selection

Simulated data were analyzed using the open-source FRETBursts Python package (Version 0.8.3).^64^ Initially, the background was independently calculated for every 360 s period of simulation (although the background doesn’t change in the simulations, this was done to keep the data analysis pipeline as close to standard protocol as possible). Following the background calculation, a burst search was performed, selecting bursts with a minimum threshold of 6 times the background signal in the donor and acceptor channels, a minimum total burst size of 60 photons in the donor and acceptor channels. The effect of varying the background multiplier and minimum burst size on the mean photon emission rate in the donor and acceptor channels is shown in Figure S5.

##### Dynamic analysis

BVA^16^ and FRET-2CDE^18^ were calculated using implementations included in the FRETBursts Python package. FRET-2CDE was calculated using a kernel shaped with a Laplace distribution. The burst-wise mean photon nanotime (referred to in the main text as the burst-wise fluorescence lifetime) was calculated using the ‘calc_mean_lifetime’ function within the FRETBursts module ‘burstlib_ext.py’, which is imported by default. To correct for the offset of the simulated instrument response function (IRF) the mean lifetime of the IRF was subtracted from the mean lifetime of the burst.

##### FRET weighted dynamic signal

The FRET weighted dynamic signal (FRET_WDS_) was created to provide a single-value summary of the deviation of the data from its expected static baseline. First, E values were grouped into bins of width 0.05 spanning −0.1 to 1.1, with a minimum of 10 events required per bin for analysis. Each bin was then compared to the corresponding expected value for a static sample, and the mean deviation was calculated as a weighted average across bins, using the number of events in each bin as the weight.

In the case of the BVA and FRET-2CDE, the FRET_WDS_ was calculated against a static control (Figure S2B). FRET_WDS_ is defined as:

$${\text{FRET}}_{\text{WDS}}=\frac{\sum_{i=1}^{N}w_{i}\cdot \left(s_{i}-c_{i}\right)}{\sum_{i=1}^{N}w_{i}}$$

where s_i_ and c_i_ denote the dynamic signal in the i-th FRET bin of the sample and control respectively. w_i_ is the statistical weight assigned to that bin and is proportional to the number of bursts within the bin.

For the burst-wise fluorescence lifetime, the FRET_WDS_ was calculated against the static line using the following:

$${\text{FRET}}_{\text{WDS}}=\frac{\sum_{i=1}^{N}w_{i}\cdot \left(\frac{E_{i}+\tau_{i}-1}{\sqrt{2}}\right)}{\sum_{i=1}^{N}w_{i}}$$

where E_i_ is the FRET efficiency of the i-th burst and τ_i_ is the normalized fluorescence lifetime for the same burst.

##### Upper and lower bounds of k_obs_

To calculate the rates at which a dynamic analysis method was able to provide a clear dynamic signal, the average FRET_WDS_ was first calculated for individual datapoints from a 'baseline' region, defined as simulations with a k_obs(sim)_ ≤ 2 s^−1^ (shaded gray area in the figures). Here, three independent replicates of each simulation were analyzed. Data points within this baseline region were assumed to appear static on the timescale of our observation time (i.e., the diffusion time of the molecule through the confocal spot), an assumption supported by the fact that this region is at least an order of magnitude from where any rise in FRET_WDS_ is observed. The mean (μ_baseline_) and standard deviation (σ_baseline_) were calculated. The baseline mean was used to normalize FRET_WDS_ by:

$$\text{Normalised}\;{\text{FRET}}_{\text{WDS}}=\frac{{\text{FRET}}_{\text{WDS}}-\mu_{\text{baseline}}}{{\text{FRET}}_{\text{WDS},\max\;}-\mu_{\text{baseline}}}$$

This normalization allows comparison between different methods on a relative scale and helps visualize small changes for simulations with small ΔE values, where absolute changes in FRET_WDS_ are small compared to large ΔE values. A multiplier of 6 was used on the σ_baseline_ to define a detection threshold for dynamics, scaled by the same normalizer:

$$\text{Threshold}=\frac{6\cdot \sigma_{\text{baseline}}}{{\text{FRET}}_{\text{WDS},\max\;}-\mu_{\text{baseline}}\;}$$

This threshold is plotted as a horizontal black dashed line in the figures. The lower (k_low_) and upper (k_high_) bounds for k_obs_ mark the start and end of the unbroken region where the mean normalized FRET_WDS_ exceeds the threshold. To minimize spurious threshold crossings due to noise, k_low_ was defined as the first point of a run of at least 5 consecutive points above the threshold. k_high_ was defined as the last point of that same unbroken run. Any single point dropping below the threshold terminates the detected range. If the signal remains above the threshold for all remaining k_obs_ values (i.e., it never drops back below), no k_high_ is recorded, indicating that the method can report on dynamics faster than the highest k_obs_ simulated.

#### Photon-by-photon hidden Markov modeling

Photon-by-photon hidden Markov modeling (H^2^MM)^19^ was performed using the burstH2MM Python package (Version 0.1.7)^37^ within the FRETBursts environment (Version 0.8.3).^64^ The H^2^MM analysis was performed on the D_em_ and A_em_ photon stream for a two-state model. The k_obs_ derived from the H^2^MM (k_obs(H2MM)_) was calculated by

$$k_{\text{obs}\left(H^{2}\text{MM}\right)}={\;k}_{12\left(H^{2}\text{MM}\right)}+{\;k}_{21\left(H^{2}\text{MM}\right)}$$

A subset of simulated data were selected for model selection analysis due to the computational demand of the state fitting, comprising 10 FRET state pairs (ΔE = 0.05–1.0), 7 observed exchange timescales spanning ∼0.4–4 10^5^ s^−1^ (one per order of magnitude), 5 equilibrium constants (K = 0.05, 0.2, 1.0, 5.0, 20.0), and 7 burst counts (*N* = 250-5,000). Models with 1–4 states were fitted to replicate 1 of the simulated data. The accuracy of BIC′ and ICL for two-state model selection was then evaluated as a function of burst count, timescale, ΔE and K (Figures S7A and S7B).

### Quantification and statistical analysis

Each simulated condition was produced as three independent replicates, generated from separate Brownian motion and photon trajectory simulations with different starting seeds (seeds = 1, 2 and 3). Unless stated otherwise, summary values for the BVA, FRET-2CDE and burst-wise fluorescence lifetime methods are reported as the mean of these three replicates, so n refers to the number of independent simulation replicates rather than to individual bursts. The photon-by-photon hidden Markov modeling was computationally intensive and was therefore performed on replicate 1 of a reduced parameter subset; the sample sizes for that analysis are given in the method details.

Where error bars are shown they represent one standard deviation across the three replicates. Boxplots show the interquartile range (25th to 75th percentile) with the median marked as a line, whiskers extending to the most extreme values within 1.5 times the interquartile range, and any further points plotted individually as outliers. For the detection limit analyses, mean values are reported only where all three replicates exceeded the detection threshold, with conditions not meeting this criterion omitted to avoid misrepresentation by outliers.

## Supplemental information

Document S1. Figures S1–S7 and Tables S1–S2

Document S2. Article plus supplemental information
