# Supplementary material for: Benchmarking dynamic analysis methods in diffusion-based single-molecule FRET
Source: Cell Rep Methods. 2026 Jul 14;6(9):101529. doi: 10.1016/j.crmeth.2026.101529 (PMC13615502; doi:10.1016/j.crmeth.2026.101529)
Supplement: Supplementary file 1 [file mmc1.pdf]

Cell Reports Methods, Volume *6*

Supplemental information

Benchmarking dynamic analysis methods in diffusion-based single-molecule FRET

Joel A. Crossley

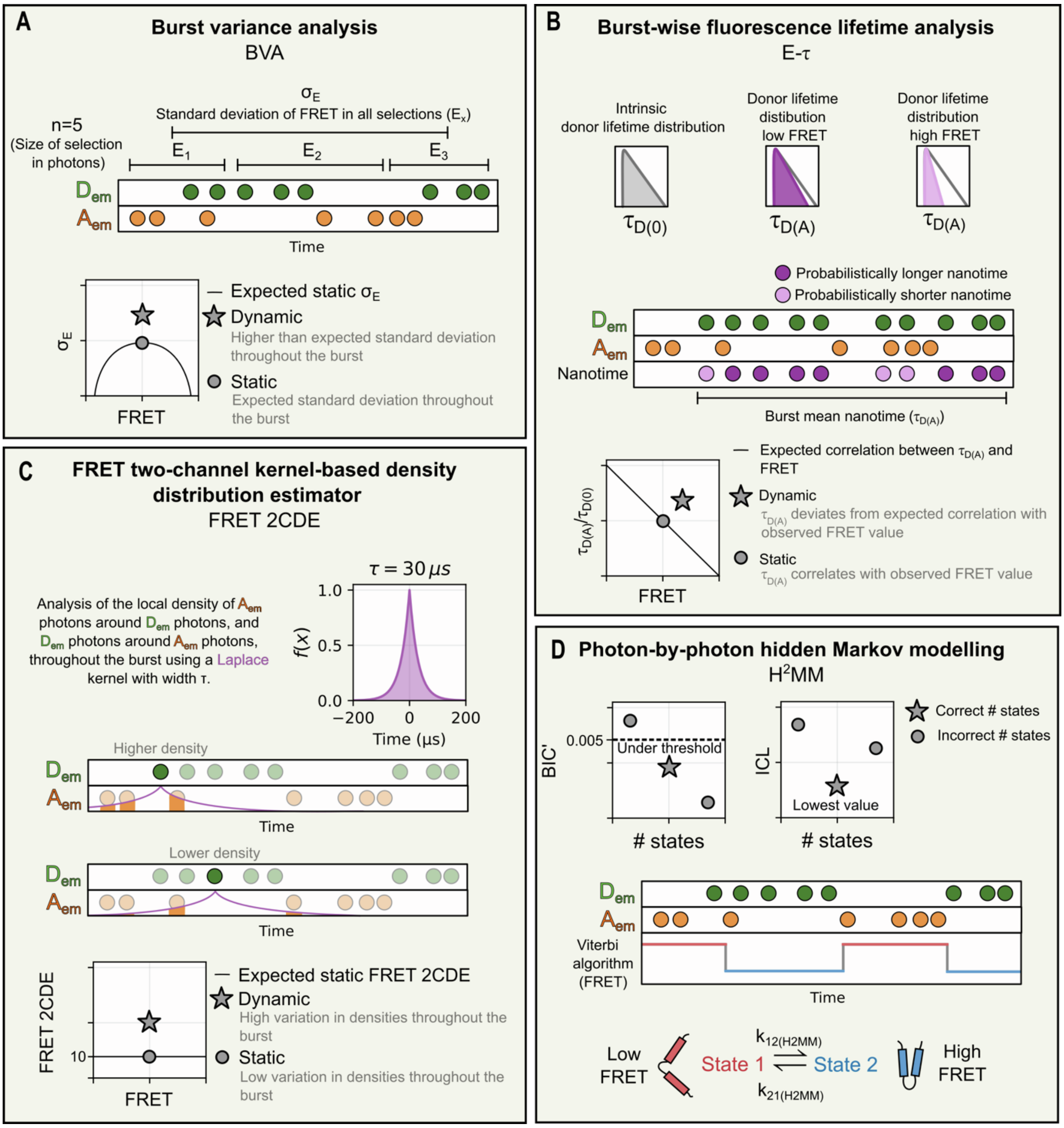

Figure S1, Related to Table 1. Schematic overview of commonly used methods to detect and quantify conformational dynamics in diffusion-based smFRET experiments. This figure is intended only to provide a brief conceptual overview of how each approach works and does not capture all experimental or analytical details. References for each method are provided in Main Text **Table 1**, where readers are directed for more detail. **(A)** Burst variance analysis, **(B)** Burst-wise lifetime analysis, **(C)** FRET two-channel kernel-based density distribution estimator and **(D)** photon-by-photon hidden Markov modeling.

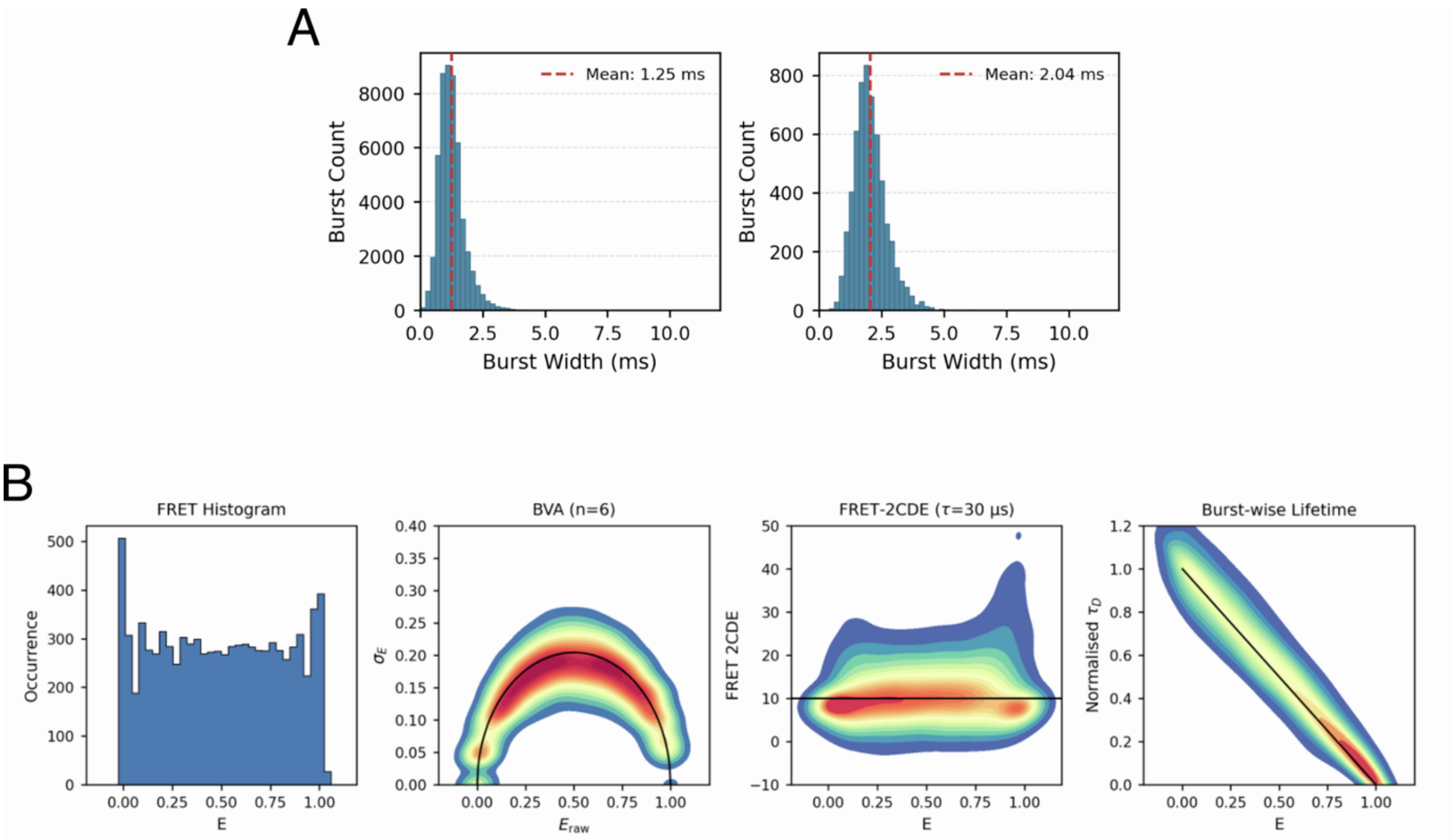

Figure S2, Related to Figure 2. FRET burst-width histograms and static FRET control. **(A)** FRET burst-width histograms before (left) and after (right) main burst selection. Before selection (F = 6, minimum burst size of 10 photons), the mean burst time is 1.25 ms (red dashed line); after selection (F = 6, minimum burst size of 60 photons), the mean burst time is 2.04 ms (red dashed line). This shift to longer mean burst times is expected because, for a fixed molecular brightness, larger burst-size thresholds preferentially select longer bursts with more detected photons. **(B)** Static FRET control (see **STAR Methods** for details). From left to right: FRET histogram, BVA (n = 6), FRET-2CDE (τ = 30 µs) and the burst-wise lifetime. Burst search parameters matched those of the main analysis (F = 6, minimum burst size of 60 photons).

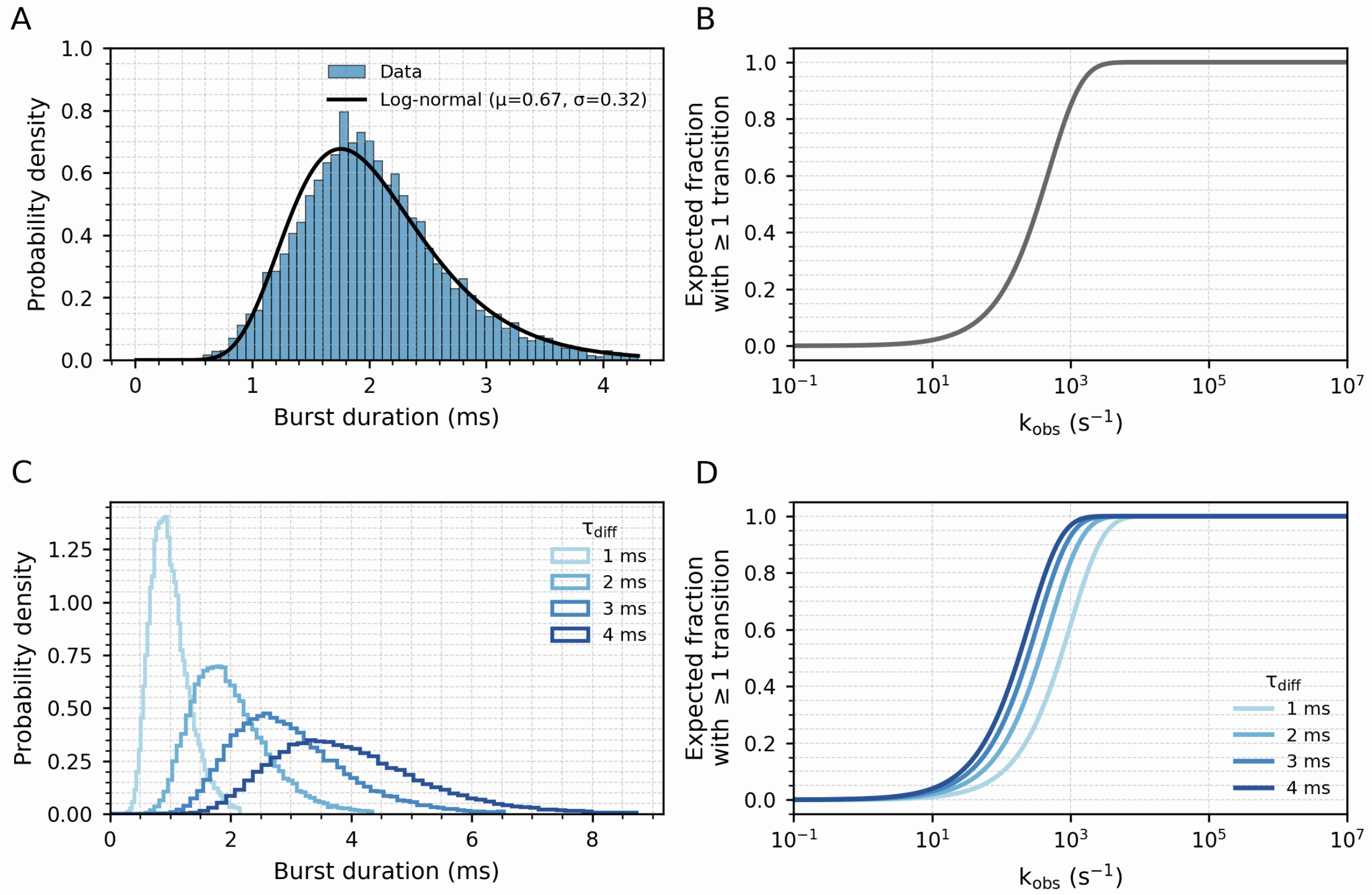

Figure S3, Related to Figure 3. Burst duration statistics in relation to the probability of observing transitions. **(A)** Burst duration histogram showing a log-normal fit (black line, μ = 0.67, σ = 0.32) to the data. **(B)** The expected fraction of bursts containing at least one transition given a specific k_{obs}, calculated by a Monte Carlo approach (50,000 steps) from the panel A data. **(C)** Generated burst duration probability density functions given the fitted parameters in panel A to represent means of 1, 2, 3 and 4 ms (50,000 samples in each). **(D)** Estimation of the fraction of molecules to contain at least one transition from the distributions in panel C using Monte Carlo sampling (50,000 steps). The k_{obs} at which 5% of bursts are expected to contain a transition for 1, 2, 3 and 4 ms are 53, 27, 18 and 13 s-1 respectively. Burst widths were obtained from the simulated smFRET data file with FRET states E = 0.25 and 0.75, K = 1 and k_{obs} = 0.25 s-1 (replicate 1), selected using F = 6 and burst size threshold = 60.

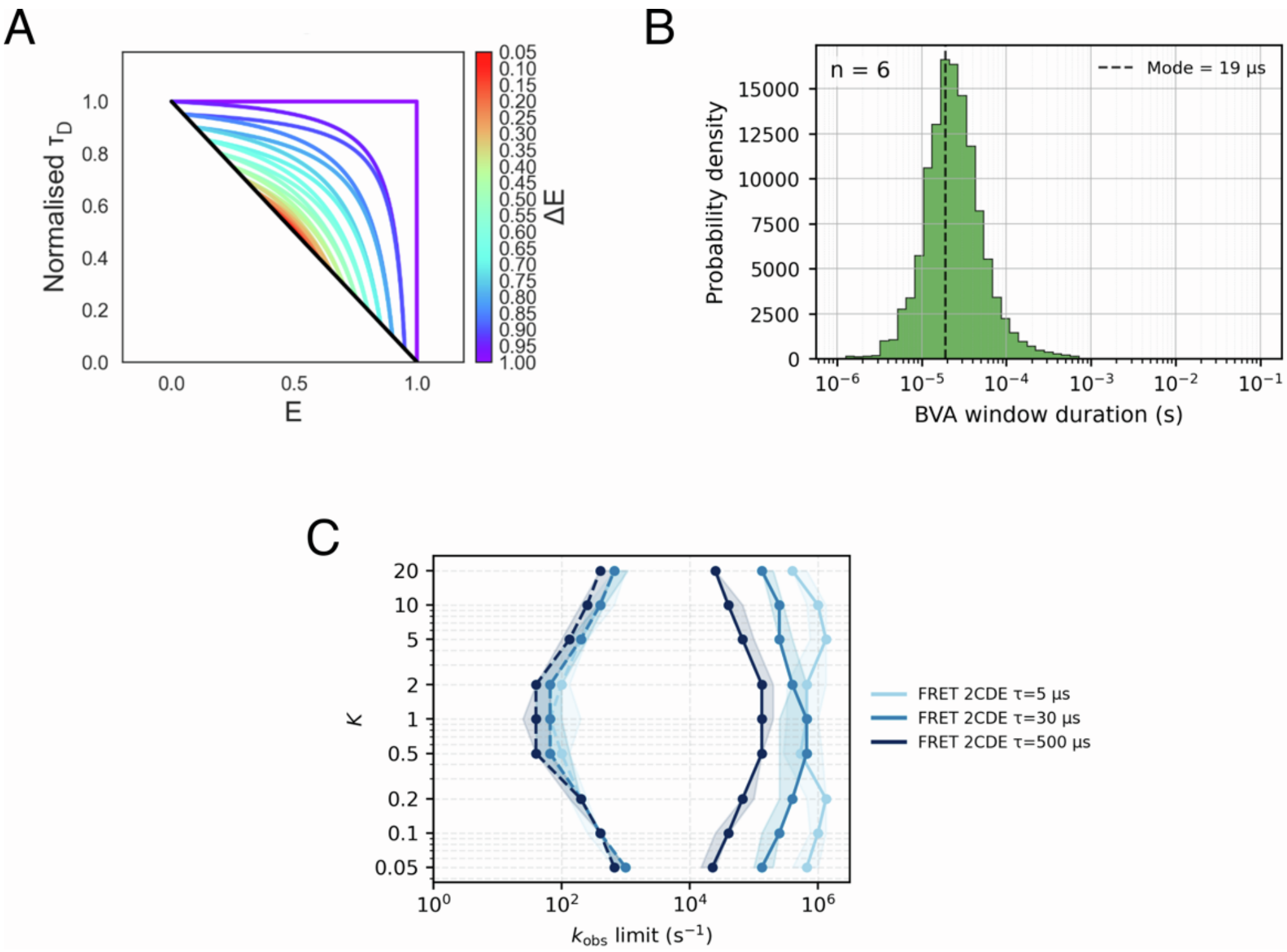

Figure S4, Related to Figure 3. Expected dynamic FRET line, BVA window durations, and FRET-2CDE detection limits. **(A)** The expected dynamic line between different FRET states for a range of ΔE values centered around E = 0.50. **(B)** Distribution of BVA photon-chunk durations in time for a photon chunk size of n = 6, with the mode centered around 19 µs. **(C)** Effect of kernel width on FRET-2CDE detection windows across equilibrium constants: lower (k_{low}; dashed) and upper (k_{high}; solid) bounds of k_{obs} as a function of K for three FRET-2CDE kernel widths (τ = 5, 30 and 500 µs) with ΔE ≥ 0.20 and burst number of 5500. Shaded regions represent the spread across ΔE values.

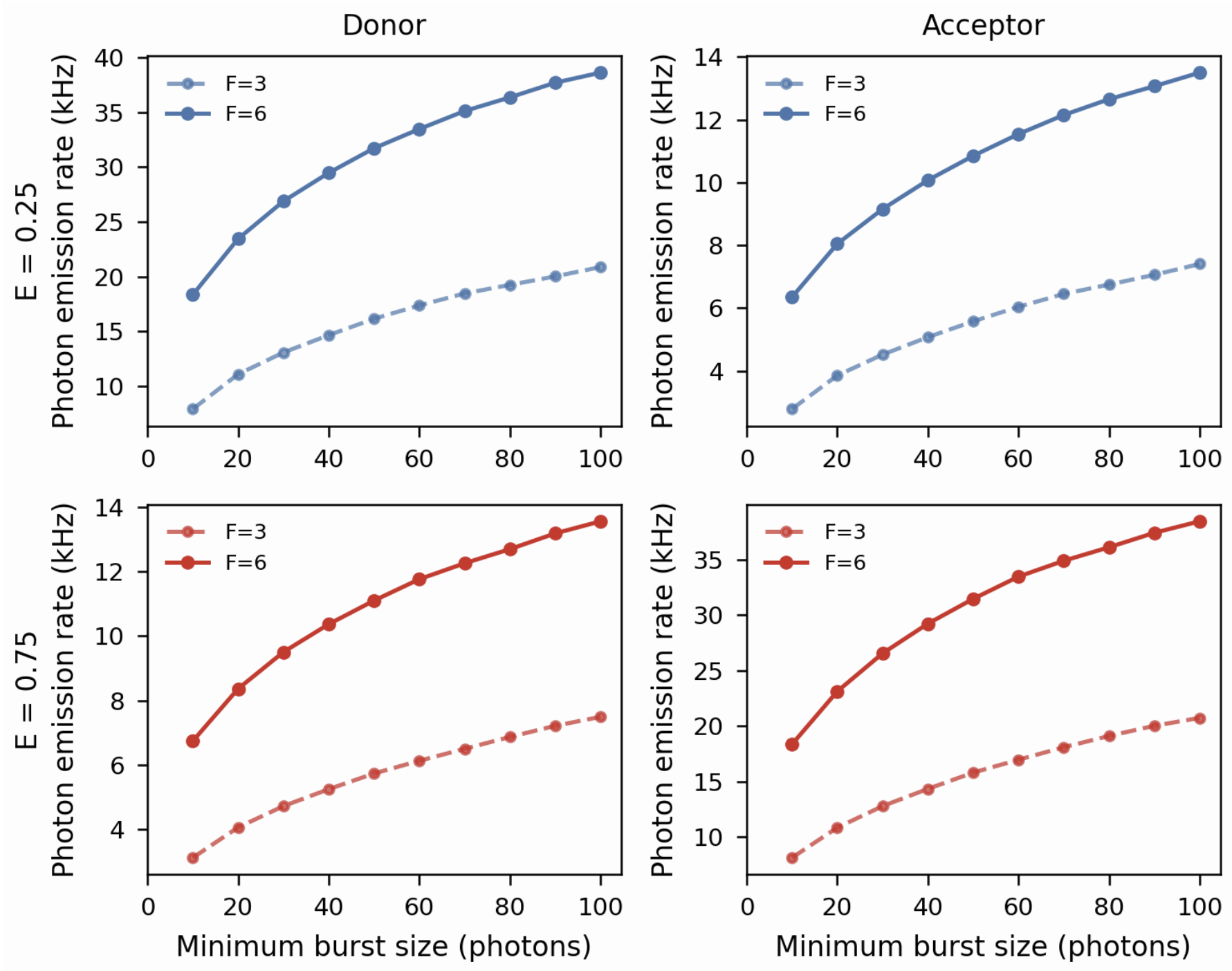

Figure S5, Related to Figure 4. Effect of burst selection parameters on mean photon emission rates. Mean photon emission rate in the donor (left) and acceptor (right) channels as a function of minimum burst size for two background multiplier thresholds (F = 3, dashed; F = 6, solid) at E = 0.25 (top, blue) and E = 0.75 (bottom, red). Higher background multipliers and larger minimum burst sizes preferentially retain bursts that pass closer to the center of the PSF, increasing the mean detected photon rate per burst.

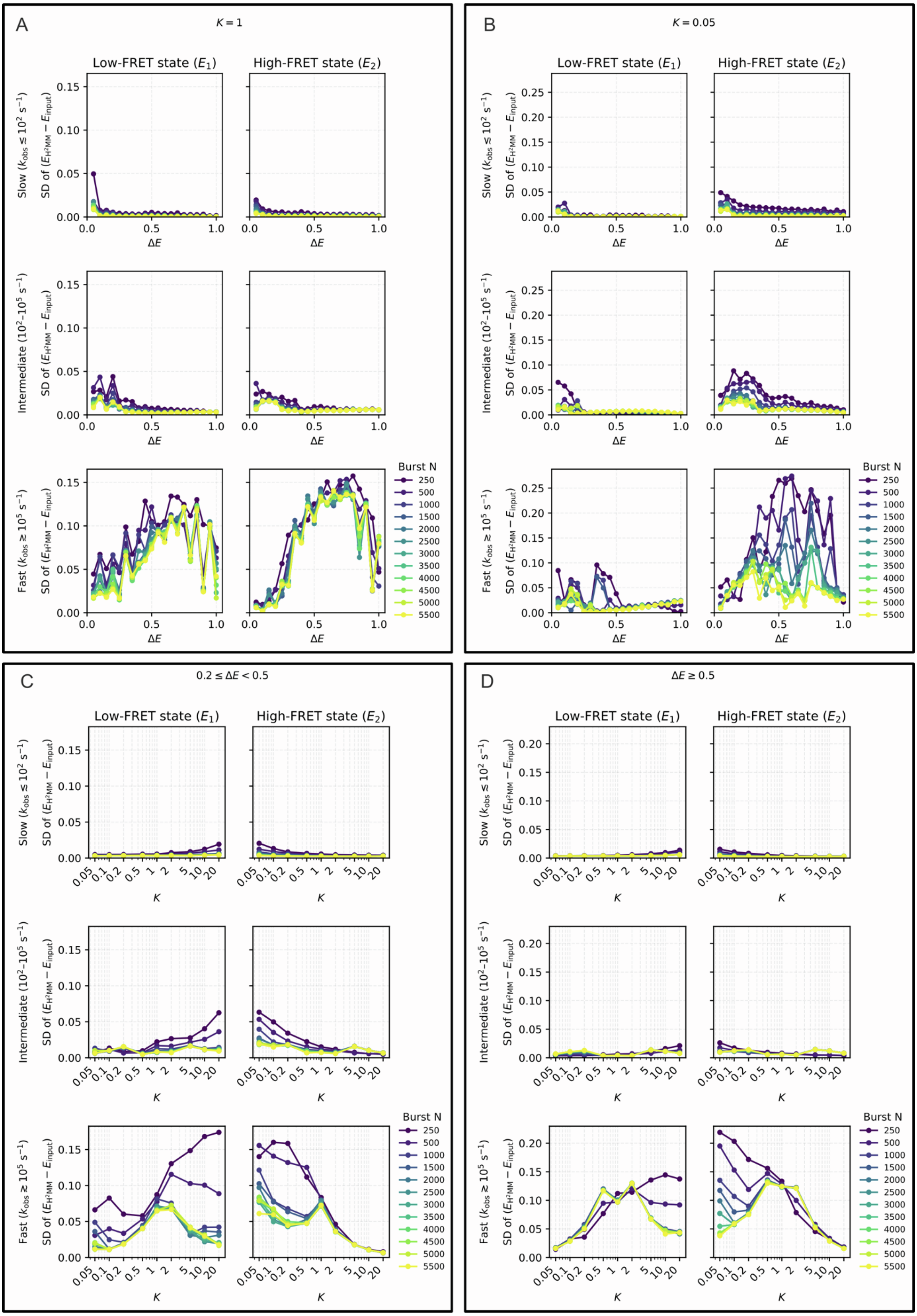

Figure S6, Related to Figure 5. Accuracy of H2MM-retrieved FRET state efficiencies as a function of ΔE, the equilibrium constant (K), kinetic regime, and burst count. The standard deviation of the difference between

H2MM-estimated and ground-truth FRET efficiencies is shown for the low-FRET state (E1, left column) and the high-FRET state (E2, right column) across slow ($k_{obs} \leq 10^2$ s^{-1}; top row), intermediate (10^2 – 10^5 s^{-1}; middle row) and fast ($k_{obs} \geq 10^5$ s^{-1}; bottom row) exchange regimes. In **(A)** K = 1 and **(B)** K = 0.05, the standard deviation is plotted as a function of FRET state separation (ΔE); in **(C)** closely spaced FRET states ($0.20 \leq \Delta E < 0.50$) and **(D)** well-separated FRET states ($\Delta E \geq 0.50$), it is plotted as a function of the equilibrium constant (K). Lines are colored by the number of FRET bursts used in the analysis.

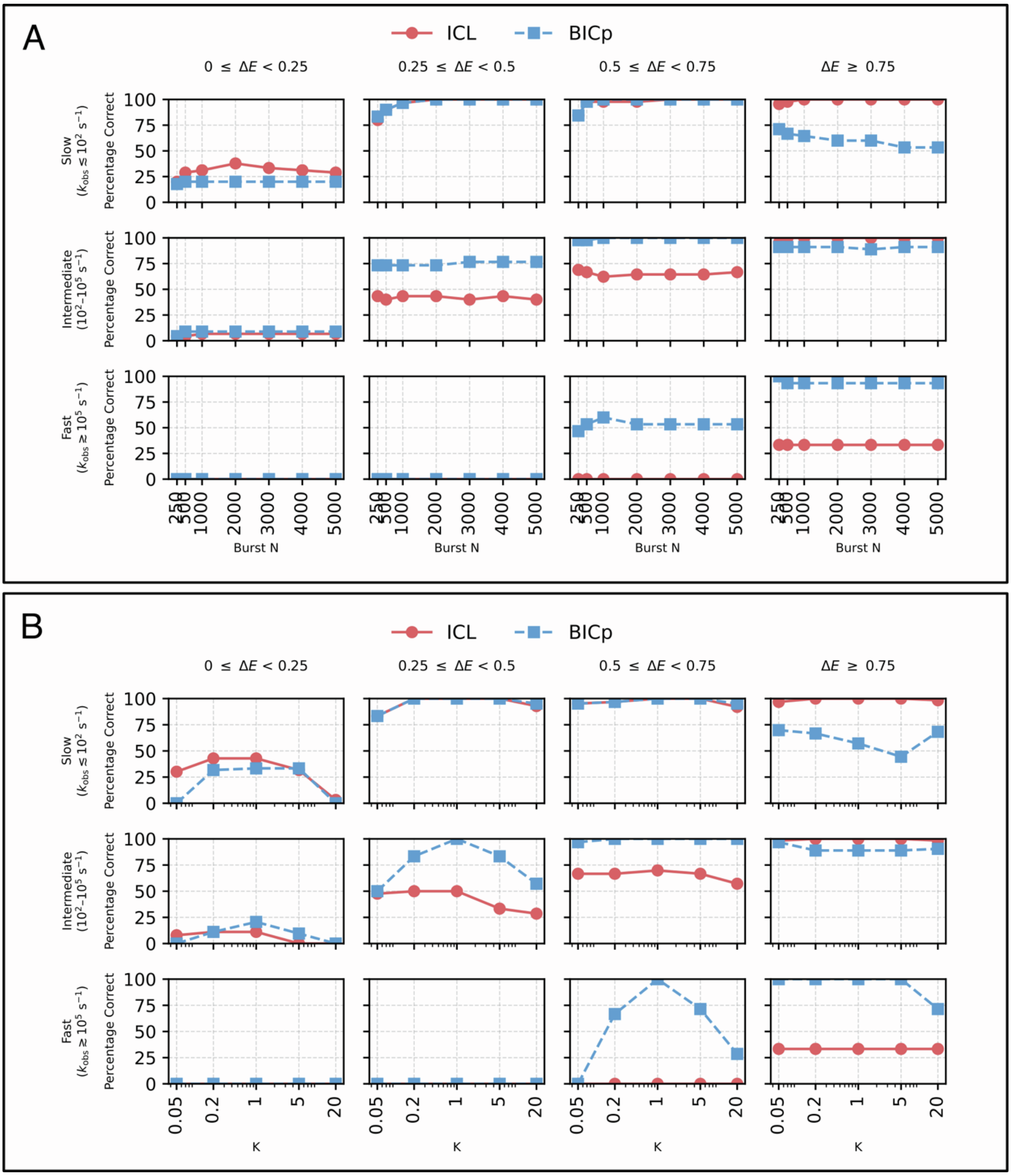

Figure S7, Related to Figure 5. Model selection accuracy of ICL and BIC′ (BICp) as a function of burst count, the equilibrium constant (K), kinetic regime and FRET state separation. The percentage of simulated datasets for which each criterion correctly identified the number of FRET states is plotted for the integrated complete likelihood (ICL, red circles) and the modified Bayesian information criterion (BIC′, blue squares), against **(A)** the number of bursts and **(B)** the equilibrium constant (K). For each simulated dataset, the two-state ground-truth model was compared against alternative one-, three- and four-state models, and the criterion was scored as correct when it preferred the two-state model. Rows correspond to the observed kinetic regime: slow ($k_{obs} \leq 10^2$ s^{-1}), intermediate ($10^2 < k_{obs} < 10^5$ s^{-1}) and fast ($k_{obs} \geq 10^5$ s^{-1}). Columns correspond to increasing FRET state separation (ΔE).

**Low FRET state (E)**	**High FRET state (E)**	**ΔE**
0.00	1.00	1.00
0.00	0.95	0.95
0.05	0.95	0.90
0.05	0.90	0.85
0.10	0.90	0.80
0.10	0.85	0.75
0.15	0.85	0.70
0.15	0.80	0.65
0.20	0.80	0.60
0.20	0.75	0.55
0.25	0.75	0.50
0.25	0.70	0.45
0.30	0.70	0.40
0.30	0.65	0.35
0.35	0.65	0.30
0.35	0.60	0.25
0.40	0.60	0.20
0.40	0.55	0.15
0.45	0.55	0.10
0.45	0.50	0.05

Table S1, Related to STAR Methods. The low and high FRET state combinations used to define the ΔE conditions in this manuscript.

Scenario	Histogram appearance	Qualitative detection	Quantitative rate extraction (H^2MM)	Key interpretation
**No prior expectation of exchange rate**	Multiple populations or a single peak; no prior expectation of the kinetic regime.	Start with BVA and FRET-2CDE; both flag dynamics by deviation above the static line. For $\Delta E \geq 0.15$, detection spans ~10^2-10^6 s^{-1} (ΔE-dependent).<br>With TCSPC hardware, E-τ extends detection beyond ~10^6 s^{-1} (limited by donor lifetime, not inter-photon interval); requires $\Delta E \geq 0.25$ for states near E = 0.50.<br>All three methods apply to the same raw data and probe overlapping but distinct timescales.	Where qualitative methods indicate dynamics, H^2MM can recover k_{obs} within ~0.5 log_{10} across a ΔE-dependent window (~10^2-10^6 s^{-1}).<br>Agreement between ICL and BIC′ provides confidence in the recovered state number; disagreement should prompt consideration of the likely kinetic regime and ΔE before favoring one over the other. Individual k_{12} and k_{21} are further limited by ΔE and K; rates for sparsely populated states become unreliable at extreme K.	No dynamic signal above the static line in BVA and FRET-2CDE does not confirm a static system; dynamics may be slower than the observation window or faster than the inter-photon rate.<br>See slow and fast exchange scenarios.
**Slow exchange ($k_{obs} \lesssim 10^2$ s^{-1})**	Well-separated FRET populations, minimal bridging density. BVA, FRET-2CDE and E-τ all lie on the static baseline.	None detectable. Transitions too infrequent for the ~1 ms observation window. Consistent with a static system or slow exchange on a timescale of seconds or longer.	H^2MM lower bound is ~10^2-10^3 s^{-1} (ΔE-dependent), exceeding rates in this regime.<br>For state extraction ICL preferred for $\Delta E \geq 0.75$, where it approaches 100% accuracy while BIC′ plateaus at 45-70%. For $0.25 \leq \Delta E < 0.75$ the two criteria are comparable. Both perform poorly at $\Delta E < 0.25$.	Distinct populations do not imply a static system; exchange may be much slower than the ~1 ms observation window. Longer trajectories (surface-tethered, ABEL trap) or RASP is needed to access slower kinetics.
**Intermediate exchange (~10^2-10^6 s^{-1})**	Bridging density between populations from partial averaging during diffusion; a fraction of molecules appear at intermediate E values.	Optimal regime for all qualitative methods. BVA and FRET-2CDE require $\Delta E \geq 0.15$; for $\Delta E \geq 0.50$, detection spans ~4 orders of magnitude ($k_{low} \approx 20$ s^{-1} to $k_{high} \approx 5\cdot10^5$ s^{-1}). E-τ requires $\Delta E \geq 0.25$ near E = 0.50; sensitivity improves away from 0.50.<br>Detection stable for K = 0.2-5. Beyond this, the rate window narrows from ~4 to ~2 orders of magnitude. E-τ shows directional sensitivity to population bias and fails at strong asymmetry (K ≈ 0.05).	Most reliable regime. k_{obs} recoverable within ~0.5 log_{10} across ~1-4 orders of magnitude (ΔE-dependent).<br>Burst count does not substantially affect the lower (~10^2-10^3 s^{-1}) or upper (~10^4-10^6 s^{-1}) accuracy bounds. Upper bound decreases as K deviates from unity; k_{12} and k_{21} for sparsely populated states become unrecoverable at extreme K.<br>BIC′ preferred for $0.25 \leq \Delta E < 0.75$, where it markedly outperforms ICL. For $\Delta E \geq 0.75$ the two are comparable, with ICL marginally ahead. Neither is reliable at $\Delta E < 0.25$.	Optimal regime for burst-based dynamic analysis. Intermediate E bursts should not be taken as evidence for dynamics or additional states without verification by H^2MM or sub-ensemble lifetime fitting.<br>Extracted k_{12} and k_{21} should not be treated as quantitatively reliable without considering ΔE and K.
**Fast exchange ($k_{obs} \gtrsim 10^6$ s^{-1})**	Single dynamically averaged population near the population-weighted mean. BVA and FRET-2CDE lie on the static line despite dynamics being present.	E-τ only. Sensitive to donor lifetime changes rather than inter-photon timing; requires TCSPC.<br>BVA and FRET-2CDE indistinguishable from a static system; cannot infer absence of dynamics.	H^2MM upper bound ~10^5-10^6 s^{-1} (ΔE-dependent). Model-selection accuracy reduced; BIC′ preferred across all ΔE; ICL accuracy falls to ~33% in this regime while BIC′ recovers the correct state number in most cases.<br>Rates should be treated with caution; validate with sub-ensemble lifetime fitting where possible.	A single population cannot distinguish static from rapid exchange without lifetime-resolved detection or kinetic modeling.<br>Where fast exchange is suspected (e.g. disordered or unfolded proteins), TCSPC is a requirement.

**Closely spaced FRET states ($\Delta E < 0.25$)**	Partially or fully overlapping populations regardless of kinetic regime; difficult to distinguish states or confirm dynamics visually.	BVA and FRET-2CDE detect dynamics for $\Delta E \geq 0.15$ but over a narrow window ($k_{low} \approx 10^3$, $k_{high} \approx 4 \cdot 10^4$ s^{-1}) versus ~4 orders of magnitude for well-separated states.<br>E-τ unreliable near $E \approx 0.50$ at $\Delta E < 0.25$; sensitivity improves away from 0.50.	Rate range reduced to ~1-2 orders of magnitude; both lower and upper bounds adversely affected.<br>Both ICL and BIC′ perform poorly and recovered state numbers should be treated with caution. ICL holds a modest advantage at slow timescales; neither is reliable at intermediate or fast timescales.	Increasing ΔE via alternative labeling positions substantially improves both qualitative and quantitative sensitivity. Where relabeling is infeasible, increasing burst count partially compensates, particularly for k_{low} of qualitative methods.
**Low burst count (< ~2,000 FRET bursts)**	Any of the above. Dataset contains < ~2,000 FRET-only bursts after removing incomplete labeling, photobleaching and blinking artifacts.	All methods applicable but slow-dynamics sensitivity reduced: k_{low} increases ~1 order of magnitude (~10^2 to ~10^3 s^{-1}) below 2,000 bursts.<br>k_{high} largely unaffected for $\Delta E \geq 0.50$; both bounds worsen for closely spaced states.	Lower and upper accuracy bounds not substantially affected by burst count under conditions tested. Larger datasets nevertheless advisable where dynamics are of primary interest.<br>Burst count has minimal impact on either criterion above ~1,000 bursts; the choice between ICL and BIC′ should instead be guided by the kinetic regime and ΔE.	Total detected bursts ≠ usable FRET-only bursts. Incompletely labeled molecules and events with photobleaching or blinking must be removed; usable count is always lower than the total. Direct excitation of the acceptor (ALEX/PIE) facilitates this filtering and is highly recommended.

Table S2, Related to Figure 6. Practical guide for selecting analysis approaches in diffusion-based smFRET experiments. Recommended approaches are given for six common experimental scenarios defined by the appearance of the FRET efficiency histogram and experimental conditions. All rate boundaries are approximate and derived from benchmarking simulations ($\Delta E \geq 0.30$, $K = 1$ and > 2,000 FRET bursts unless otherwise stated).
